# Carbon Dots: Synthesis, Properties and Applications

**DOI:** 10.3390/nano11123419

**Published:** 2021-12-16

**Authors:** Lin Cui, Xin Ren, Mengtao Sun, Haiyan Liu, Lixin Xia

**Affiliations:** 1 Liaoning Key Laboratory of Chemical Additive Synthesis and Separation, Yingkou Institute of Technology, Yingkou 115014, China; s20200786@xs.ustb.edu.cn; 2 School of Mathematics and Physics, University of Science and Technology Beijing, Beijing 100083, China; 3 Yuanyang Branch Department, Beijing Jingshan School, Beijing 100040, China; 4 International Department, Beijing No. 12 High School, Beijing 100071, China; shoushidarenxin@163.com

**Keywords:** nanomaterial, carbon dots, biomedicine, photocatalysis

## Abstract

Carbon dots (CDs) are known as the rising star of carbon-based nanomaterials and, by virtue of their unique structure and fascinating properties, they have attracted considerable interest in different fields such as biological sensing, drug delivery, photodynamic therapy, photocatalysis, and solar cells in recent years. Particularly, the outstanding electronic and optical properties of the CDs have attracted increasing attention in biomedical and photocatalytic applications owing to their low toxicity, biocompatibility, excellent photostability, tunable fluorescence, outstanding efficient up-converted photoluminescence behavior, and photo-induced electron transfer ability. This article reviews recent progress on the synthesis routes and optical properties of CDs as well as biomedical and photocatalytic applications. Furthermore, we discuss an outlook on future and potential development of the CDs based biosensor, biological dye, biological vehicle, and photocatalysts in this booming research field.

## 1. Introduction

As zero-dimension carbon-based nanomaterials with ultrafine sizes below 10 nm, carbon dots (CDs) are promising candidates for numerous applications [1,2,3,4,5]. CDs are a newly developed type of carbon nanomaterial and composed of discrete, quasi-spherical nanoparticles. They were first discovered from the components of fluorescent nanoparticles during single-walled carbon nanotubes purification in 2004. Until 2006, these carbon nanoparticles were named as “carbon quantum dots” by Sun et al. who proposed a route to synthesize CDs by simple surface passivation and chemical modification for enhancing fluorescence emission [6]. After that, CDs have received extensive and significant attention owing to their unique structure and fascinating properties in different fields. As one of the new allotropes of carbon, CDs show many remarkable advantages, such as low cytotoxicity [7], good biocompatibility [8], stable chemical inertness [9], efficient light harvesting [9] and outstanding photoinduced electron transfer [10], thus making them promising candidates for various applications in biosensors [11,12,13], bioimaging [14,15], optoelectronic devices [16,17], solar cells, etc. [18,19].

**Figure 1:**
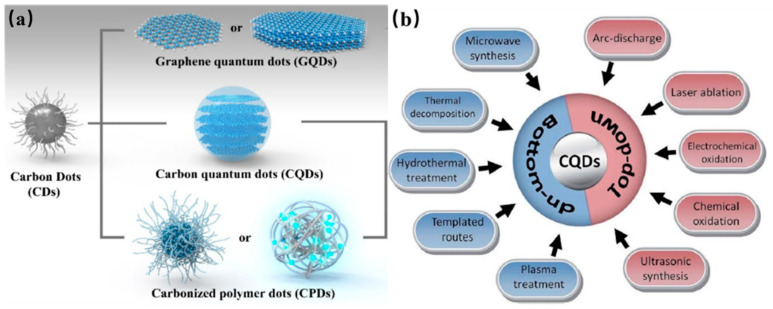
(**a**) Carbon dots including graphene quantum dots, carbon nanodots and polymer dots [28]. Copyright 2020, American Chemical Society. (**b**) Carbon dots synthesized from “top-down” and “bottom-up” approaches [29]. Copyright 2017, Royal Society of Chemistry.

Carbon dots, broadly regarded as small carbon nanoparticles in aqueous or other suspensions, are generally categorized into three types, namely, graphene quantum dots (GQDs), carbon nanodots (CNDs), and carbonized polymeric dots (CPDs) as shown in Figure 1a. Most CDs are commonly comprised of the sp^2^/sp^3^ hybridized carbon core with surface functional groups. For instance, GQDs is composed of mono- or multi-layer nano-sized graphite and surface/edge functional groups or interlayer defects, which are anisotropic with lateral dimensions larger than their height, and their optical properties are mainly dominated by the size of π-conjugated domains and the surface/edge structures [20,21,22]. Different from the GQDs, CQDs and CPDs possess typically spherical core connected with surface groups. The spherical core of CQDs exhibit multiple-layer graphite structures, and their photoluminescence (PL) emission properties are mainly dominated by the intrinsic state luminescence and the quantum confinement effect of size. CPDs are the hybrid nanostructures, consisting of aggregated/cross-linked carbon cores and polymer chains shells, and their optical properties of CPDs are mainly dominated by the molecular state and cross-link structure. The different structures of the CDs are affected by various synthesis routes which can be generally classified into “bottom-up” strategies and “top-down” strategies [23,24,25,26,27,28,29] as shown in Figure 1b. Hence, the surface functional groups of the CDs can be changed by utilizing different synthesis routes, which achieves tunable light emission. In particular, the modified surface moieties of the CDs facilitate to expand the light utilization range from different states of emissive trap owing to their efficient up-converted photoluminescence behavior, indicating that efficient visible-light-responsive CD can be designed in full spectrum of sunlight. Furthermore, the light harvesting ability of the CDs-based composites is beneficial to photoexcited electron transfer for improving photocatalytic efficiency.

Although the synthesis routes, properties and applications of the CDs have been systematically summarized elsewhere, there are few dedicated reviews with emphases on the synthesis routes, properties and applications of the CDs [30,31,32,33]. Hence, aiming at a distinct perspective, we will compile a general review on the recent progress of the CDs. We intend to spotlight the latest advances in the CDs research, focus on synthesis, PL mechanism, and applications of CDs in this feature review [34,35,36,37,38]. Specifically, we first mainly summarize the synthesis routes to produce the CDs from different resources in especially low cost, and environmentally friendly natural biomass in Section 2. And then, properties of the CDs which mainly include optical, electronic and catalytic properties and the fluorescence mechanisms are discussed in Section 3. In Section 4, we discuss the fundamental roles and potential development of the CDs based photocatalysts in photocatalytic systems. Finally, we hope this review can offer valuable insight and critical insights for the investigation of the CDs, and further inspire more exciting work on the CD-based biosensors and photocatalysts in practical application.

## 2. Synthesis of the CDs

The various routes have been proposed to fabricate the CDs with desired properties for a particular application during the last decade. Generally, these well-established synthesis strategies of CDs are classified into “top-down” and “bottom-up”. The top-down strategies include ultrasonic synthesis, chemical exfoliation, electrochemical oxidation, arc-discharge, and laser ablation. In general, GQDs, 2D nanoparticles, are obtained by “top-down” strategy from the exfoliation and cutting of macroscopic carbon structures with the obvious graphene lattices, such as graphite powder, carbon black, activated carbon, carbon nanotubes, carbon soot, and carbon fibers. Top-down approaches generally require long processing times, harsh reaction conditions and expensive materials and equipment [20,21,22]. This strategy is suitable for mass production. In contrast, CQDs and CPDs, both are 3D nanoparticles with spherical core, are typically synthesized from the bottom-up approaches. The CQDs and CPDs are produced via polymerization of molecular precursors, such as glucose, sucrose, and citric acid, through microwave pyrolysis, solvothermal reactions, plasma treatment and chemical vapor deposition, which show fewer defects and high controllability [20]. Here, we will elaborate the main methods for CDs synthesis, size control and surface moieties by treatment of confined pyrolysis and modification. Surface properties of CDs could be optimized during preparation or further treatment for the selected applications.

### 2.1. Top-Down Approaches

#### 2.1.1. Chemical Exfoliation

**Figure 2:**
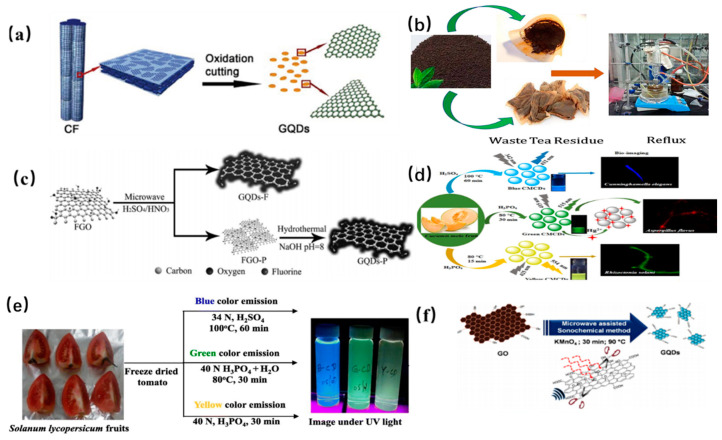
Representation of chemical exfoliation of various carbon precursors dots. (**a**) Carbon fiber [40]. Copyright 2012, American Chemical Society. (**b**) Green tea leaf residue [42]. Copyright 2019, Elsevier. (**c**) FGO [44]. Copyright 2015, John Wiley and Sons. (**d**) Muskmelon fruit [46]. Copyright 2019, American Chemical Society. (**e**) Tomato [47]. Copyright 2019, Elsevier. (**f**) GO [48]. Copyright 2017, American Chemical Society.

Chemical exfoliation is a facile and convenient method for mass production of high-quality CDs without complicated devices. The precursor carbon materials (carbon fibers, graphene oxide, carbon nanotubes) are cleaved by strong acids or oxidizing agents. Mao and coworkers fabricated for the first time fluorescent GQDs with different sizes from candle soot by using HNO_3_ under a relatively high temperature in 2007 [39]. Afterwards, Peng et al. prepared GQDs through a chemical exfoliation of carbon fibers by H_2_SO_4_ and HNO_3_ as shown in Figure 2a [40]. The as-prepared GQDs with different sizes ranges show yellow, green and blue PL emission under different stirring temperatures, respectively, indicating that the successful preparation of GQDs can be achieved by chemical cleavage of carbon fiber. Later, some low-cost materials were considered as precursor to prepare GQDs under strong acid conditions. Ye and his coworkers prepared hexagonal GQDs with a size range of 3–6 nm by the chemical cleavage of coal with the crystalline carbon. Subsequently, Zhao et al. utilized asphaltene to fabricate GQDs with excitation-dependent PL emission. However, it is a tedious process to further purify the product by the removal of excess H_2_SO_4_, increasing the overall synthesis cost [41].

Later, Gunjal et al. prepared N-doped GQDs with high PL quantum yield by concentrated H_2_SO_4_ cleavage of green tea leaf residue as shown in Figure 2b [42]. The obtained N doped GQDs could be used as turn off sensor for the detection of gefitinib [43]. Sun et al. proposed an acid vapor cutting route to prepare Fluorinated GQDs by using fluorinated graphene oxide derived carbon with a low volume of H_2_SO_4_/HNO_3_ as shown in Figure 2c [44]. The GQDs product was easily obtained without any laborious purification process via in situ filtration. It is notable to mention that the nitric acid cleavage of the sp^2^ domain of MOF determines the formation of GQDs. Soni et al. prepared N, S co-doped CDs with a bright green PL emission by the triflic acidic cleavage of palm shell powder. The sizes of GQDs could be tuned by changing the length of the amino chains [45]. And it should be noted that PL quantum yield and up-conversion properties of GQDs could also be tuned by further surface passivation. Actually, citric acid was widely utilized as carbon source for the synthesis of CQDs. For example, Wang et al. utilized citric acid as a coordinating solvent to prepare CQDs at 240 °C. The resultant CQDs with an average diameter of 0.9 nm have a high quantum yield of 47% and can be easily fabricated into fluorescent film, which indicated that the CQD may be applied in the fields of the catalysis, photovoltaic and optical-power limiting. Desai et al. utilized sulfuric and phosphoric acid as an oxidizing agent to prepare CQDs derived from muskmelon fruit as shown in Figure 2d. The as-prepared CQDs with high quantum yield showed intense PL emission, further indicating that the peripheral moieties on the surface of GQDs can influence their PL emission [46]. Moreover, the obtained GQD with the longer PL life time have great potential application in the biological probing. 

In fact, except for strong acids, various powerful oxidants were also used in the preparation of CDs. For example, Kailasa and coworkers utilized H_2_SO_4_ as an oxidant to prepare blue-, green- and yellow-color fluorescent GQDs by chemically oxidizing tomato as shown in Figure 2e [47]. The obtained GQDs were evaluated for assaying Fe^3+^ ions and exhibited low limit of detection up to 0.016 μM. Fe^3+^ ion was quantified in iron supplements and biofluids [47]. Later, Nair et al. reported that GO can be oxidized by KMnO_4_, yielding GQDs with a high fluorescence QY [48]. Although one pot acid-free route is highly efficient for synthesis of GQDs, the purification process of the final product was tedious and complex for the removal of oxidant as shown in Figure 2f. Similarly, Zhu et al. utilized hydrogen peroxide as mild oxidant to oxidize GO to fabricate GQDs with the help of W_18_O_49_ nanowires and the size of the obtained GQDs is dependent on the concentration between W_18_O_49_ and H_2_O_2_ [49]. Compared to acid-based oxidizing agents, further purification is not necessary when mild oxidants are utilized in the synthesis process of GQDs. The as-prepared GQDs could be directly applied in the field of bioimaging, indicating that the acid-free strategy is simple and environmentally friendly.

#### 2.1.2. Laser Ablation

**Figure 3:**
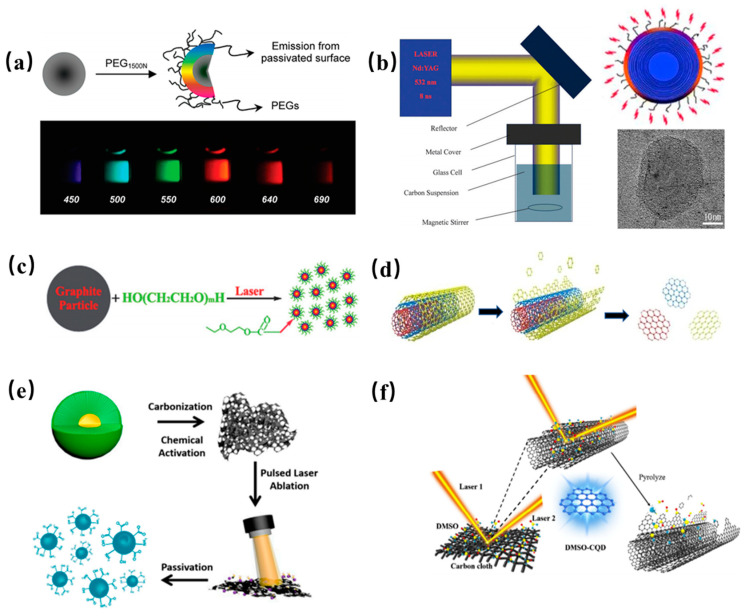
Schematic diagram of (**a**) Fabrication of GQDs by the laser ablation of graphite [6]. Copyright 2006, American Chemical Society. (**b**) Laser passivation of carbon particles to synthesize CQDs [50]. Copyright 2011, Royal Society of Chemistry. (**c**) Laser irradiation of a suspension of carbon powders [51]. Copyright 2009, Royal Society of Chemistry. (**d**) multi-wall carbon nanotube to prepare CQDs [52]. Copyright 2016, Springer Nature. (**e**) Preparation process of the N-CQDs derived from the Platanus biomass [53]. Copyright 2019, MDPI. (**f**) Ultrafast and highly efficient preparation of DMSO-CQD by dual-beam pulsed laser ablation [54]. Copyright 2020, Elsevier.

As a unique and promising synthesis route, laser ablation has been applied in the preparation of CDs owning to advantages of short period and simple operation. Sun et al. first demonstrated laser ablation synthesis of GQDs from graphite (see Figure 3a) [6]. Li et al. prepared GQDs with visible, stable and tunable PL performance by laser rapid passivation of carbon particles, and demonstrated that passivation by laser irradiation has an important influence on the origin of PL as shown in Figure 3b [50]. Similarly, Hu et al. proposed a facile route to synthesize fluorescent carbon nanoparticles by laser ablation of carbon powders suspension in organic solvent as shown in Figure 3c [51]. The surface of the resultant GQDs can be modified by selecting appropriate solvents. Thus, PL emission of the GQDs was tunable by changing their surface functional groups, which was attributed to carboxylate ligands on the surface of the GQDs. Kang proposed a new and remarkably rapid preparation route to synthesize GQDs from multi-wall carbon nanotube with excellent optoelectronic properties as shown in Figure 3d [52]. The synthesized GQDs show stable and blue PL emission. The PL quantum yield is as high as 12%, which were suitable for optoelectronic applications. Ren et al. prepared NM-CQDs by laser ablation of the carbonized Platanus biomass in the formamide solution [53]. The obtained CQDs showed dual-wavelength photoluminescence (PL) emission as seen in Figure 3e. Subsequently, Ren’s group proposed an ultrafast method to fabricate homogeneous CQDs by dual-beam pulsed laser ablation, which improved the preparation efficiency [54]. Schematic diagram of synthesis of the CQDs from carbon fiber is presented in Figure 3f.

#### 2.1.3. Ultrasonic-Assisted Treatment

**Figure 4:**
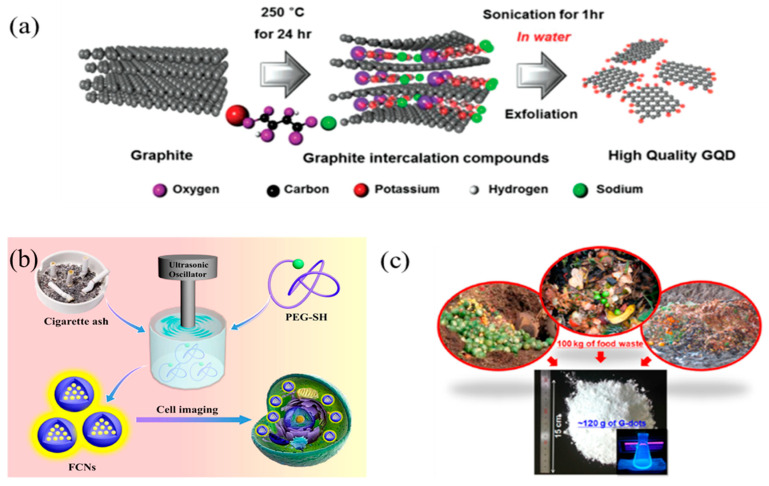
Schematic diagram of (**a**) Preparative strategy of GQDs [57]. Copyright 2014, John Wiley and Sons Inc. (**b**) Fabrication of polymer-functionalized CQDs by ultrasonic-assisted treatment [58]. Copyright 2018, Elsevier. (**c**) Synthesis and application of CDs derived from food waste [59]. Copyright 2014, American Chemical Society.

It is acknowledged that ultrasonic-assisted method has the advantages of low cost and simple operation for the preparation of CDs. Alternate high-pressure waves and low-pressure were generated in the ultrasound process, which results in the formation and collapse of small bubbles in liquid. Thus, macroscopic carbon materials were cut into nanoscale CDs by strong hydrodynamic shear forces derived from the cavitation of small bubbles. Generally, researchers prepared the CDs with different properties by simply adjusting the ultrasonic power, reaction time and the ratio of carbon sources and solvents. Zhuo et al. reported the preparation of GQDs by ultrasonic exfoliation of graphene for the first time [55]. Since then, many researchers utilized the ultrasonic-assisted treatment to prepare GQDs from different carbon materials including graphite, MWCNTs, GO and carbon fiber in either aqueous solution or organic solvent [56]. Subsequently, Song and his co-workers prepared GQDs from the aqueous dispersion of graphite and potassium sodium tartrate by ultrasonic assisted treatment (see in Figure 4a) [57]. The obtained GQDs were in the range of 1–5 nm in diameter and showed blue luminescent emission. Interestingly, heteroatom-doped GQDs were also fabricated by ultrasonic approach. Zhao et al. synthesized the chlorine-doped GQDs from the chlorinated CF precursor by direct ultrasonic exfoliation approach as shown in Figure 4b [58]. In addition to carbon sources, other carbon-containing waste materials were also used in the preparation of CQDs. In a typical experiment, Park et al. first synthesized water-soluble CQDs from the food waste-derived carbon source by a simple ultrasonic irradiation treatment as shown in Figure 4c [59]. About 120 g CDs with an average diameter of 2–4 nm can be produced from 100 kg mixture of ethanol and food waste. The as-prepared CDs have advantages of good PL properties, low cytotoxicity and high photostability for in vitro bioimaging. 

### 2.2. Bottom-up Approaches

#### 2.2.1. Microwave Synthesis

**Figure 5:**
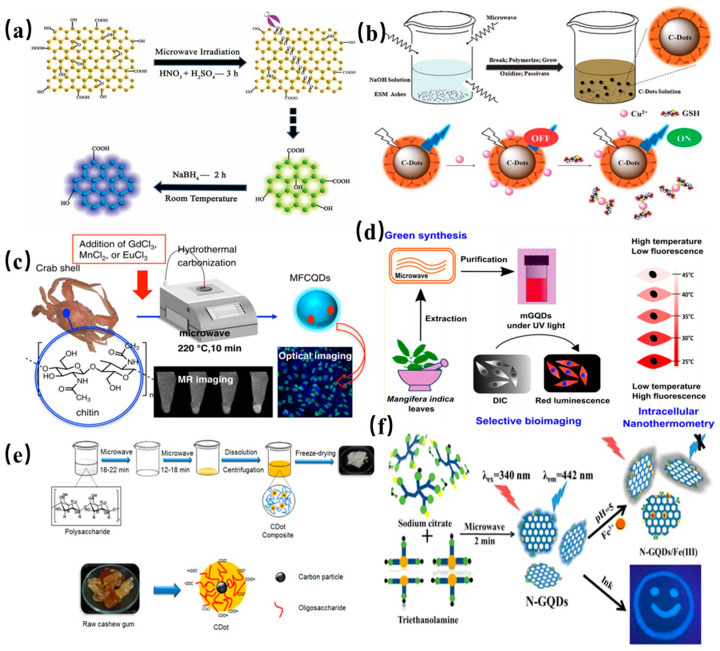
Schematic diagram of (**a**) Preparation route for gGQDs and bGQDs [60]. Copyright 2012, John Wiley and Sons. (**b**) Preparation process and application of the fluorescence CDs from protein-rich eggshell membranes by microwave-assisted approaches [61]. Copyright 2012, Royal Society of Chemistry. (**c**) Synthesis of the CDs from crab shell [62]. Copyright 2017, American Chemical Society. (**d**) Preparation and application of the soluble CDs from mango leaves [63]. Copyright 2017, American Chemical Society. (**e**) Preparation of the fluorescent CDs composites from raw cashew gum [64]. Copyright 2015, Sociedade Brasileira de Química. (**f**) Synthesis of fluorescent N-GQDs from triethanolamine and sodium citrate [65]. Copyright 2019, American Chemical Society.

A green, cost-effective microwave assisted strategy is widely employed to synthesize the CDs in less time. Microwave irradiation can provide uniform heat for formation of CDs. Li et al. synthesized green fluorescent GQDs by microwave-assisted chemical cleavage of GO sheets under acidic conditions for the first time as shown in Figure 5a [60]. Several epoxy moieties on the surface of GO sheets can be cracked under microwave treatment. The obtained GQDs with single-layer have an average diameter of 4.5 nm, and can be further designed to the blue fluorescent GQDs by reduction of surface radical moieties. Wang and coworkers reported a simple one-pot microwave-assisted approach to fabricate the water-soluble CDs from protein-rich eggshell membranes [61]. The obtained CQDs show excellent fluorescence emission with a quantum yield of about 14% as well as the capability of simultaneous determination of Cu^2+^ and glutathione. A synthetic scheme of CQDs through a microwave-assisted method is presented in Figure 5b. As shown in Figure 5c, Yao et al. proposed a new route to produce novel fluorescent CQDs from transition-metal ions and crab shell by microwave-assisted hydrothermal. The as-prepared Gd @ CQDs display high stability against pH and NaCl concentration, indicating that Gd @ CQDs have the potential application in drug delivery [62]. In another work, CDs were obtained from *Mangifera indica* leaves by a simple microwave-assisted hydrothermal (see Figure 5d) [63]. The obtained CDs possessed good biocompatibility and high photostability, which were used as an intracellular temperature sensor. Wang et al. proposed a two-step microwave heating process to produce CDs from raw cashew gum [64]. The obtained CQDs show good biocompatibility and low cytotoxicity, and thus were utilized in live-cell imaging as shown in Figure 5e. Subsequently, Ren et al. reported the preparation of N-doped GQDs with a size of 5.6 nm in diameter by microwave-assisted heat as shown in Figure 5f. The resultant N-GQDs show intense and stable blue fluorescence emission with a quantum yield of 8% and were applied in metal ion detection [65]. Ricardo et al. proposed a microwave-assisted synthesis route to prepare blue-emitting CQDs and investigated PL emission properties [66]. They found that green-emitting molecular fluorophores were produced in synthesis process, which can mask the PL emission of the blue-emitting CQDs. It should be emphasized that these fluorophores and the carbon dots do not behave as separated species with individual emission in the same solution. Instead, a hybrid luminescence by their interaction is observed. These phenomena indicate that excited state properties and reactivity are different than just the sum of their individual properties. Fluorescent impurities originating from the synthesis process created a significant setback to investigation on PL of CQDs.

#### 2.2.2. Hydrothermal Method

**Figure 6:**
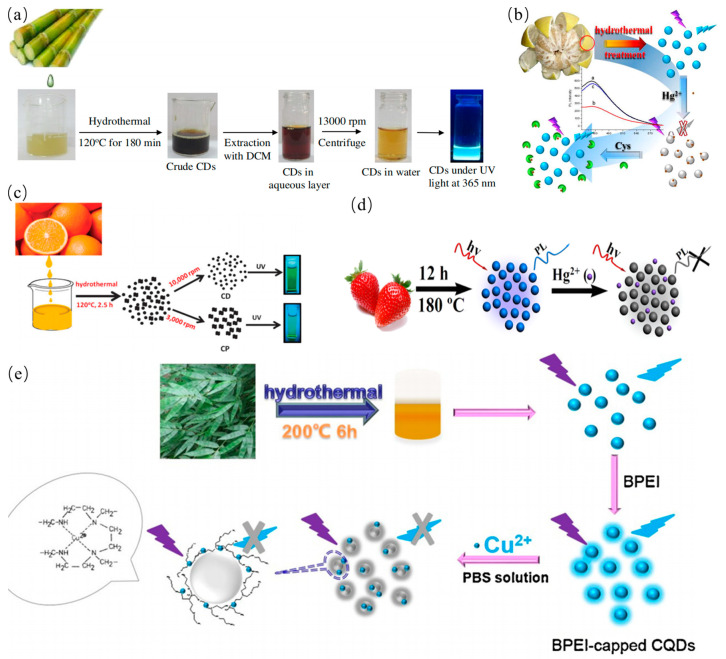
Schematic diagram of (**a**) Preparation process and application of the fluorescence CDs from *saccharum officinarum juice* [77]. Copyright 2014, Elsevier. (**b**) Synthesis of the CDs derived from pomelo peel [78]. Copyright 2012, American Chemical Society. (**c**) Preparation and application of the soluble CDs from orange juice [79]. Copyright 2012, Royal Society of Chemistry. (**d**) Preparation of the fluorescent CDs from strawberry juice [80]. Copyright 2013, Royal Society of Chemistry. (**e**) Preparation and application of the CDs from bamboo leaves [81]. Copyright 2014, Elsevier.

Hydrothermal strategy for the preparation of CDs has advantages of low cost and nontoxicity. Compared to other synthetic routes, hydrothermal method is a simple approach to synthesize CQDs. In general, the water solution of mixtures was enclosed with Teflon in an oven and hydrothermally reacted at high pressure and high temperature [67,68,69,70,71,72,73]. Pan et al. reported a green route to prepare blue fluorescence CQDs by the hydrothermal method for the first time [74]. The epoxy moieties on the surface of the GO sheets were completely broken into CQDs during hydrothermal treatment. Subsequently, Zhao’s group also reported a simple and highly efficient route to prepare CQDs by hydrothermal treatment [75]. Some researchers also utilized oxidants to accelerate the hydrothermal reaction. For example, Halder et al. reported a simple hydrothermal method to prepare GQDs by adding H_2_O_2_ for accelerating exfoliation of GO sheets, indicating effective scissors of the GO sheets by H_2_O_2_ during hydrothermal reaction [76]. Meanwhile, compared to the traditional precursors, waste biomass was also seen as carbon sources. Mehta. et al. reported a plant-based hydrothermal route for green synthesis of the water-soluble fluorescent CQDs with an average size of 3 nm derived from *Saccharum officinarum* juice [77]. The as-prepared CQDs were used for selective and sensitive detection of Cu^2+^ as shown in Figure 6a. Lu et al. proposed a hydrothermal route to produce CQD with the average particle size of 4 nm from pomelo peel [78]. The obtained CQDs have quantum yield of 6.9%, which were utilized to sensitively detect the Hg^2+^ at low concentration for the analysis of lake water sample as seen in Figure 6b. Sahu et al. synthesized the CQD with size of 1.5–4.5 nm from orange juice at 120 °C for 150 min in an autoclave via one step hydrothermal method [79]. These CQDs show tunable luminescence properties and good biocompatibility as shown in Figure 6c. Moreover, Huang et al. utilized strawberry juice as resource to produce fluorescent nitrogen-doped CQDs by the simple, low cost and green solvothermal method [80]. Obviously, the as-prepared CQDs with a nitrogen content of 6.88% were used in the selective and sensitive detection of Hg^2+^ as shown in Figure 6d. Liu et al. proposed a simple and low-cost synthesis route of CQDs from bamboo leaves [81]. PL quantum yield of CDs is as high as 7.1%. The CQDs were capped with branched poly-ethylenimine via electrostatic adsorption for sensitive and selective detection of Cu^2+^ in river water as shown in Figure 6e. In plants, the CDs derived from other biomass wastes such as wheat straw, coffee grounds, onion waste, wheat bran, tobacco leaves, etc. as biomass carbon precursors are also demonstrated by hydrothermal method as shown in Table 1. Essner et al. though that the inadequacy of purification gives rise to misconceptions about the nature and characteristics of the CDs. They first prepared CQDs solution by hydrothermal and microwave routes followed by dialysis or ultrafiltration purification steps [82]. The comparison results showed the formation of molecular fluorophores contribute to a majority of the PL emission of CDs, further indicating that the fluorescent impurities must be removed for the reliable result. Therefore, we suggested that many previous studies will need to be carefully revisited using more rigorous purification protocols [83].

#### 2.2.3. Solvothermal Method

**Figure 7:**
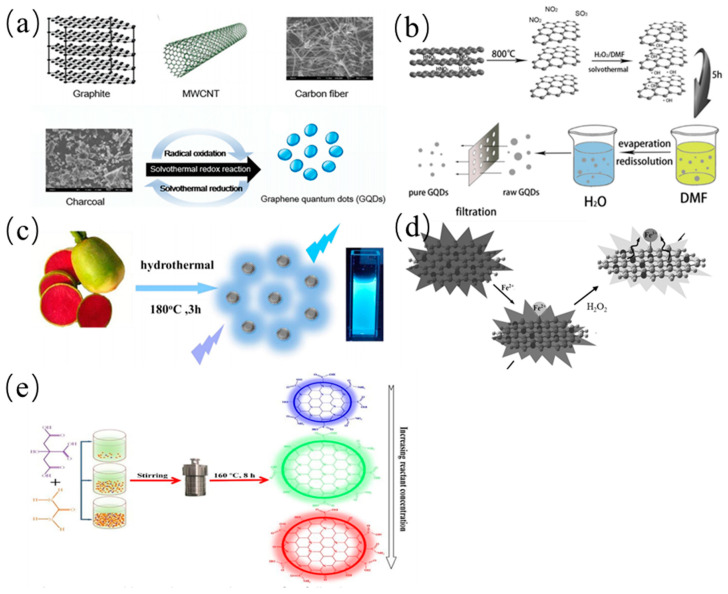
Schematic diagram of (**a**) solvothermal exfoliation of different carbon-based precursors to synthesize the fluorescence CQD [97]. Copyright 2015, Royal Society of Chemistry. (**b**) Synthesis of the CQDs from graphite in DMF solution [98]. Copyright 2018, Elsevier. (**c**) Synthesis of N-CDs from rose-heart radish [99]. Copyright 2017, Elsevier. (**d**) Schematic of the sensing mechanism for H_2_O_2_ detection based on NCQDs and Fe^2+^ [100]. Copyright 2014, John Wiley and Sons. (**e**) Synthesis mechanisms for full-color emitting CQDs [102]. Copyright 2020, Royal Society of Chemistry.

Apart from the hydrothermal fabrication, solvothermal approach for the preparation of CDs has advantages of low-cost and requirement of simple equipment [92]. Different from the hydrothermal method, water solution was replaced with one or several solvents sealed with Teflon equipped with a steel autoclave [93,94,95]. The solvent and the raw carbon source mixtures reacted at high pressure and high temperature. Zhu et al. prepared green fluorescent GQDs with a PL quantum yield of 11% in dimethyl formamide solvent by the solvothermal method [96]. The topographical height profile of S-GQDs revealed single- to bilayer-thick. Subsequently, Shin and coworker chemically oxidized natural carbon precursors by using oxone as non-acid mild oxidant as shown in Figure 7a [97]. The obtained GQDs with a high quantum yield showed a blue PL emission. Later, Tian et al. synthesized the blue fluorescent GQDs by solvothermal exfoliation of graphite with the help of mild H_2_O_2_ as presented in Figure 7b [98]. The obtained GQDs showed a high quantum yield of 15% and good photoluminescence stability in different pH conditions. Meanwhile, the low-cost biomass was also employed to synthesize the CQDs by solvothermal method. Liu and his coworkers established a green one-step solvothermal route to fabricate CQDs with the quantum yield of 5.7% from L-ascorbic acid and glycol solution in an autoclave at 160 °C for 4 h (see Figure 7c) [99]. The as-prepared CQDs exhibited a strong green fluorescent emission for cell labeling. Qian et al. proposed a simple solvothermal route to produce N-doped CQDs from CCl_4_ and diamines mixture at 200 °C [100]. After dialysis, the purified CQDs were obtained and showed the higher quantum yield than many other CQDs. The obtained CQDs with multifunctional fluorescence properties can be utilized for selective and sensitive determination of pH, Ag^+^, and Fe^3+^ in aqueous solution as shown in Figure 7d. Hence, an effective pH indicator was designed as a device for bioimaging. Moreover, Mitra et al. utilized PEG-200 as a precursor to produce homogeneous CQDs at 160 °C for 24 h [101]. The synthesized CQDs were then employed in bioimaging fields owning to their low toxicity and high photostability. The synthesized CQDs were then employed in bioimaging fields owning to their low toxicity and high photostability. Through a solvothermal method, Zheng et al. synthesized full-color emitting CQDs by changing the reactant concentrations in the reaction solvent [102], and possible synthesis mechanisms for full-color emitting CQDs is shown in Figure 7e. Similarly, He and his coworker found that versatile impurities (as luminescent components) originating from the ethanothermal process can also mask the luminescence of the CQDs [103]. For example, molecular fluorophore (8-ethoxy-3H-cyclopenta naphthalen-3-one, ECNO) were produced in the synthesis process and showed a single green and excitation-wavelength-independent emission. However, CQDs showed dual emission comprising a blue broad and a yellow narrow-band emission, and exhibited higher photo- and thermal stability than ECNO. ECNO and CQDs showed the dramatic differences in the optical behaviors, indicating that fluorescent molecular impurities can mask or even alter the properties of the obtained CQDs. 

#### 2.2.4. Pyrolysis/Carbonization

Pyrolysis is a powerful technique to fabricate the fluorescent CDs by using macroscopic carbon structures as precursors in recent years. This method offers advantages of short reaction time, low cost, easy operation, solvent-free approaches and scalable production. The four main processes including heating, dehydration, degradation and carbonization are critical factors for conversion of the organic carbon-containing substance into CQDs under high temperature. Carbon precursors are cleaved into carbon nanoparticles by using high-concentration alkali or acid in the pyrolysis process. 

**Figure 8:**
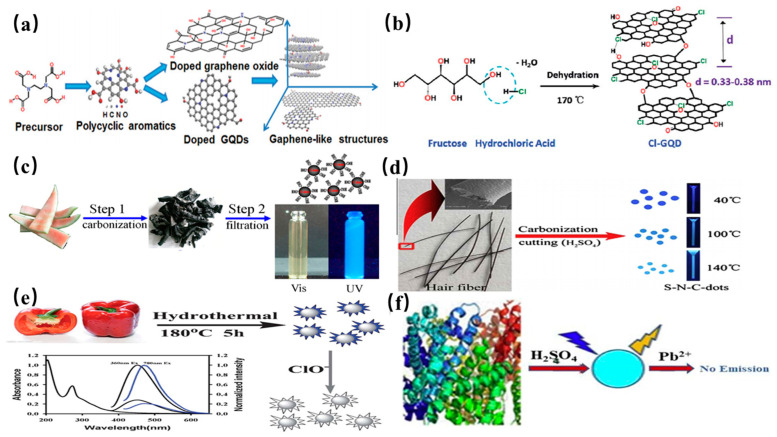
Schematic illustration of (**a**) Preparation of GQDs derived from EDTA [104]. Copyright 2015, Royal Society of Chemistry. (**b**) the preparation of Cl-GQDs from fructose and HCl [105]. Copyright 2013, Royal Society of Chemistry. (**c**) Synthesis of the water-soluble CQDs from watermelon peel [106]. Copyright 2012, Elsevier. (**d**) Formation procedure of the CQDs from hair fiber [109]. Copyright 2013, Elsevier. (**e**) Preparation of the fluorescent CDs from low-cost red pepper [110]. Copyright 2013, Royal Society of Chemistry. (**f**) Heavy metal ion (Pb (II)) detection by CQD [111]. Copyright 2013, Elsevier.

Ma et al. fabricated N-GQDs by the direct carbonization of ethylene diaminetetra acetic acid at 260–280 °C and growth mechanism of GQDs was also proposed in this study as shown in Figure 8a [104]. It is worth mentioning that synthesis of various types of CQDs were reported by ions doping. In a typical experiment, Li and co-workers fabricated Cl-GQDs by introducing HCl (see Figure 8b) [105]. The O and H groups of fructose were dehydrated and the nucleus of GQDs was formed under hydrothermal treatment [105]. Meanwhile, HCl was beneficial to accelerate the reaction and offered a Cl dopant. A green and economical synthesis route was proposed to produce high-quality fluorescent CQDs by carbonization of watermelon peel at 220 °C in ambient air for 2 h. The product was further purified to obtain the CQDs with average size of 2 nm by the three processes including ultrasonic, filtration and centrifugation treatment. The schematic illustration of the reaction process for the synthesis of the CQDs from watermelon peel is presented in Figure 8c [106]. Similarly, it was reported the synthesis of N-doped CQDs from peanut shell waste via a simple and economical carbonization method by Xue et al. The obtained CQDs exhibited excitation wavelength dependent fluorescence emission [107]. Praneerad et al. also prepared the fluorescent CQDs by carbonization of the durian peel biomass waste [108]. The obtained CQDs were utilized to construct a composite electrode which showed a much higher specific capacitance than that of the pure carbon electrode. Sun and coworkers discovered that the carbon quantum dots (CQDs) with higher N and S contents were synthesized by carbonization of hair fiber mixed with H_2_SO_4_ through sonication treatment in Figure 8d [109]. The synthesized N-S-CQDs show good luminescence stability and high solubility. Yin et al. first reported a facile carbonization approach to synthesize highly sensitive CQDs with down-and up-conversion fluorescence from the low cost sweet red pepper precursor. In this synthesis, fresh pepper was suspended in distilled water. They were then sealed in 180 °C Teflon-lined autoclave for 5 h [110]. As shown in Figure 8e, the purified CQDs were obtained after centrifugations and filtration treatment. Wee et al. synthesized CQDs with an average particle diameter of 1.2 nm from bovine serum of albumin protein by direct acid hydrolysis at 50 °C for 2 h [111]. The CQD solution has less toxicity for heavy metal ions detection as shown in Figure 8f. 

#### 2.2.5. Chemical Vapor Deposition

**Figure 9:**
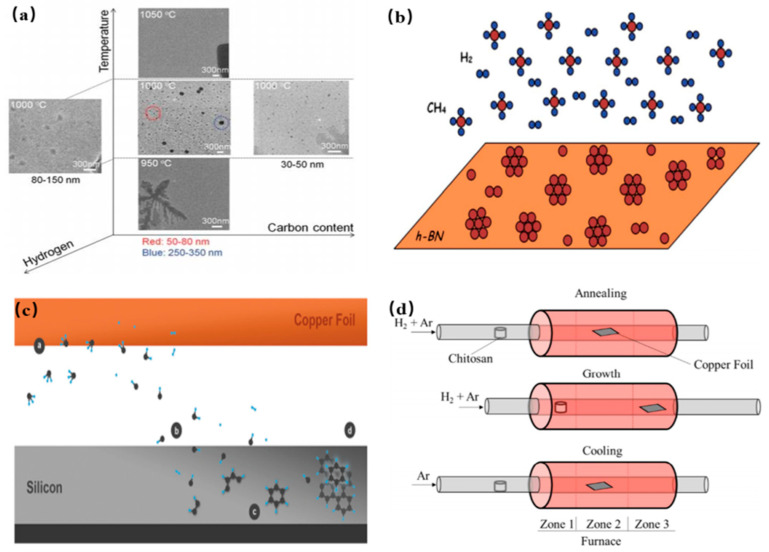
Schematic illustration of (**a**) Size control of grapheme domains and GQDs [112]. Copyright 2013, Jone Wiley and Sons. (**b**) CVD growth of GQDs on h-BN substrate [113]. Copyright 2014, Royal Society of Chemistry. (**c**) GQDs on the silicon substrate by CVD growing process [114]. Copyright 2016, John Wiley and Sons. (**d**) Synthesis of N-GQDs [115]. Copyright 2018, American Chemical Society.

As a well-known approach, chemical vapor deposition (CVD) method is used to fabricate CQDs, and has also been widely explored in recent years. In a CVD technique, the size of the ultimate product could be determined by tuning these parameters including carbon source, growth time, flow rate of the hydrogen (H_2_) and temperature of the substrate. Fan et al. first proposed chemical vapor deposition method to prepare CQDs by using methane as a carbon source as shown in Figure 9a [112]. Specifically, some oxidation groups on the surface of the copper foil were firstly cleaned by using alcohol and HCl. And then, the substrate was heated to 1000 °C in the H_2_ and Ar ambient condition. The H_2_ supply was turned off and Ar was continuously supplied for removing the H_2_ residues. Afterwards, the methane gas (CH_4_) was pumped in the furnace at a flow rate of 2 mL/min for only 3 s in an Ar environment. The size of the as-synthesized CQDs was distributed in the range of 5–15 nm and height profile thereof was 1–3 nm, indicating the successful preparation of few-layer-thick CQDs. Subsequently, Ding et al. utilized hexagonal boron nitride as a substrate to GQDs, instead of any metal catalyst as shown in Figure 9b [113]. The different numbers of GQDs were tuned by changing the ratio of Ar-: CH_4_: H_2_ gases, and exhibited thickness-dependent PL emissions in the visible region. In 2016, single crystalline GQDs were fabricated on silicon wafer, which possessed an average thickness of 1.2 nm and an average diameter of 7.5 nm as shown in Figure 9c [114]. In addition, the other sources of carbon such as chitosan was used to fabricated N-GQDs by chemical vapor deposition method. The obtained N-GQDs possessed an average diameter of 12 nm and thickness of 3 nm by the DLS analysis and the AFM height profile as shown in Figure 9d [115]. The obtained N-GQDs showed the intense PL emission in the visible region and were potentially applied in the field of nano-optoelectronic. Here, we will introduce some representative preparation methods for CDs in detail and compare their merits and demerits in Table 2.

## 3. Properties of CQDs

As we know, the CQDs are commonly classified into three types including GQDs, CQDs and CPDs, which are described in terms of “core-shell” nanostructure consisting of a nanoscale carbon core and surface functional groups. Their optical properties, electronic properties and catalytic properties are also determined by their different structures. Hereafter these properties will be discussed. 

### 3.1. Optical Properties

#### 3.1.1. UV-Absorption Property of CDs

**Figure 10:**
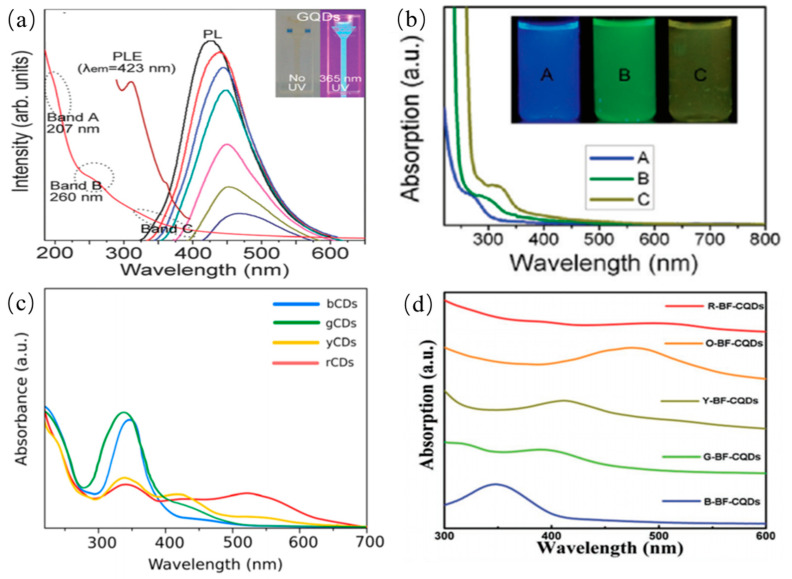
(**a**) UV, PL and PLE spectra of CQDs produced from graphite flakes [121]. Copyright 2012, Royal Society of Chemistry. (**b**) UV-vis spectra of CDs under different reaction temperatures, respectively [40]. Copyright 2012, American Chemical Society. (**c**) Absorption spectra for long wavelength emission CQDs [119]. Copyright 2017, American Chemical Society. (**d**) Absorption spectra for CQDs [120]. Copyright 2016, John Wiley and Sons.

The as-prepared CQDs from different precursors have obviously different absorption spectra in different solvents. Although CQDs possess a variety of structures, they have some similar UV-visible absorption. Here, we only summarize some common UV-visible absorption phenomenon rather than some special examples. Typically, one or more absorption peaks can be clearly observed in the UV region ranged from 260 nm to 320 nm [116,117,118,119,120,121,122]. And, a tail can extend to the overall visible region. In general, the absorption peak of the CQDs is clearly observed in the wavelength range of 220~270 nm. It can be elucidated that electrons transit from the π orbital to C=N bonds. The absorption peak lying in the region of 280~350 nm is attributed to electronic transitions from C–O or C=O bonds to π* orbital as shown in Figure 10a [121]. The absorption peak wavelength located in the range of 350~600 nm is attributed to the electron transitions of the surface functional groups of CQDs, indicating the surface chemical moieties may contribute to the absorption in the UV–visible regions. Some studies indicate the absorption peaks of CQDs red shift after the treatment of surface functional groups or adjustment of their sizes as shown in Figure 10b [40]. Even some special CQDs show long-wavelength absorption ranges of 600–800 nm, which originates from aromatic ring-containing structures as presented in Figure 10c [119]. Different from previously mentioned CDQs, the N-CQDs presented a strong excitonic absorption band owing to quantum-size effects as shown in Figure 10d [120]. To some extent, the deviations of absorption spectra of CQDs suggest differences in structures of different hybridization derivatives or the compositions.

#### 3.1.2. Fluorescence Property and PL Mechanism of CDs

**Figure 11:**
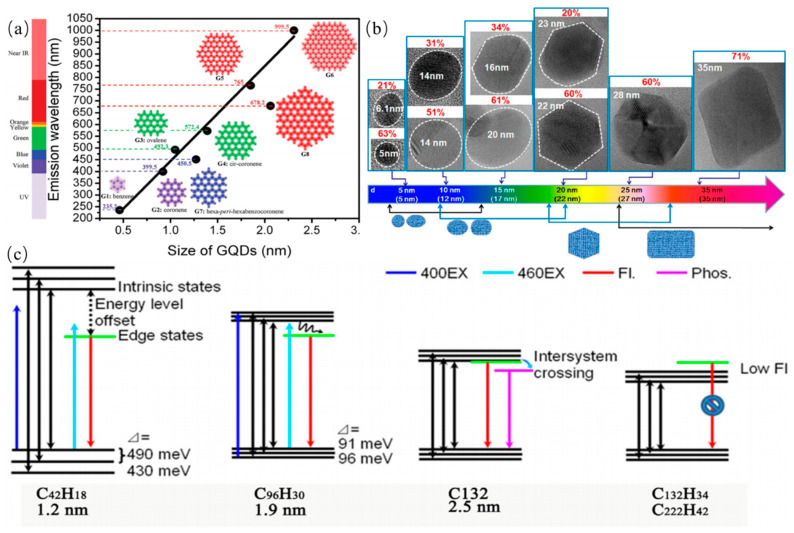
(**a**) Emission wavelength of GQDs with different sizes obtained through theoretical calculations [123]. Copyright 2014, Royal Society of Chemistry. (**b**) HRTEM images of GQDs for their major shapes and corresponding populations [124]. Copyright 2012, American Chemical Society. (**c**) The size-dependent energy levels in different H-passivated small GQDs [125]. Copyright 2014, Elsevier.

The fluorescence emission is one of the most charming features of CDs, which has been utilized in many fields. Generally, the excitation wavelength of CQDs is shorter than their PL emission wavelength, namely the Stokes PL emission. CDs have some other excellent fluorescence properties which are excitation wavelength-dependent PL emission, tunable PL emission, extraordinary up-converted PL, good fluorescence stability and efficient photobleaching resistance. PL emissions of CDs are dependent on their diversity of the structural characteristics, which are classified roughly into two categories. One is due to electrons transitions corresponding to internal factors dominated emission, which include the conjugation effect and the surface state and the synergistic effect. This model is suitable for explaining the PL of GQDs that have lattice structures or a high degree of graphitization. GQDs have certain crystallinity, with an average lattice parameter of 0.24 nm. In other words, the PL emission of the CQDs can be tuned by adjusting the size of the conjugated π-domains rather than the actual particle size since their sizes are smaller than their exciton Bohr radius. Several research groups have studied particle size dependent or independent PL of GQDs. The quantum confinement effect of GQDs was corroborated by theoretical calculations method. Sk et al. reported PL emission of pristine zigzag-edged GQDs with different diameters by Gaussian and time-dependent density-function theory (DFT) as shown in Figure 11a [123]. The wavelengths of PL emissions of GQDs with different diameters of 0.46–2.31 nm were observed across the range from deep UV to near infrared. The smallest GQDs with a diameter of 0.5 nm has a wavelength of 235.2 nm, while GQDs with a diameter of 2.31 nm has a wavelength of 999.5 nm. The PL emission of GQDs was observed in the entire visible region (400–770 nm) with the different diameters of GQDs increasing from 0.89 to 1.80 nm, which was attributed to the quantum confinement effect. This particle size dependence is consistent with the quantum confinement effect. GQDs and graphene oxide sheets, also concluded that both sp^2^ domains and defects are emission sites. The conjugation effect explains the effect of GQDs size on luminescence from the perspective of the conjugate length of the carbon core, and is generally appropriate for GQDs. The mentioned mechanisms are presented in Figure 11b,c [124,125]. The intrinsic state of GQDs also determine their PL emission behavior owing to quantum confinement effect. The conjugated π-domains are regarded as intrinsic PL center. The band gaps of GQDs decrease as aromatic rings increasing, indicating that PL emissions of GQDs can be adjusted by tuning the size of the conjugated π-domains. Kim et al. reported size-or shape- dependent PL emission. By changing size or shape of GQDs, the electronic transitions can be tuned in nanometer-sized GQDs [125]. The intense visible PL emissions can be clearly observed in a controlled fashion. These reports have also proved that the intrinsic state of GQDs with a perfect core structure plays a leading role in their PL behavior. 

**Figure 12:**
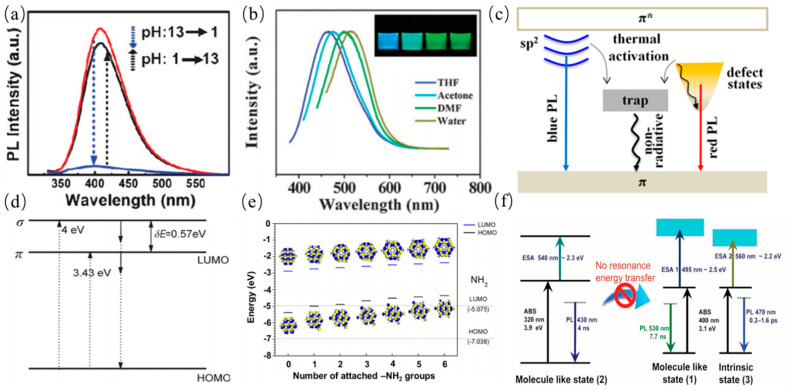
(**a**) The pH-dependent behavior of GQDs [74]. Copyright 2010, John Wiley and Sons. (**b**) The solvent-dependent PL emission behavior of GQDs [96]. Copyright 2011, Royal Society of Chemistry. (**c**) Surface/edge states emission mechanism of the GQDs [127]. Copyright 2013, American Chemical Society. (**d**) HOMO and LUMO energy levels of carbon core functionalized by the different numbers of -NH_2_ moieties [121]. Copyright 2012, Royal Society of Chemistry. (**e**) The two independent molecule-like states and a dark state existed in GQDs [128]. Copyright 2013, American Chemical Society. (**f**) the band gap of GQDs with number of attached-NH_2_ groups [129]. Copyright 2013, John Wiley and Sons.

The other is associated with surface configurations or surface/edge state including passivated surface defects, triple carbene at the zigzag edges, oxygen-containing/other functional groups [126]. These surface states play a key role in the PL emission of CQDs, whereas the diverse types of surface states can induce PL emission with different characteristics. Heteroatom doping are widely used to alter the electronic structures of GQDs. Hence it is also ascribed to the surface state effect. Zhu et al. prepared strongly fluorescent GQDs by one-step solvothermal method as shown in Figure 12a [74]. GQDs showed pH dependent PL emission which is affected under strongly acidic atmosphere owing to protonation or deprotonation of functional moieties on the surface of the carbon core-edge state. PL emission of GQDs from polystyrene foam waste was reported by Zhang et al., which is dependent on the type of organic solvent, which elucidate that different emission sites derive from different organic solvents, leading to different surface passivation upon GQDs as presented in Figure 12b [96]. Ding and coworkers proposed a hydrothermal method to prepare a series of GQDs with similar size distributions [8]. These GQDs show the surface oxidation degrees dependent PL emission which can be observed in the wavelength range of 440 to 625 nm, determining further the application of GQDs in different fields. Xu et al. synthesized GQDs by the microwave-assisted hydrothermal method. The independent PL emission of GQDs was also observed as shown in Figure 12c, which was attributed to the presence of a self-passivated layer, leading to insensitivity of graphene domains to actual size [127]. Lin et al. discovered energy gap of GQDs with zigzag edges was smaller than those with armchair edges [121]. The zigzag sites of GQDs possess probably a triplet carbene state described as *σ*^1^*π*^1^, instead of a carbyne singlet ground state as presented in Figure 12d. The corresponding credible evidences are protonation and deprotonation of the zigzag sites which can quench and recover PL emission behavior of GQDs under acidic or alkaline conditions, respectively, suggesting that PL emission was originated from the triple carbene states instead of the quantum confinement effects. In addition, hybridization of the amine moieties and carbon core can tune PL emission, which was attributed to the modified electronic structure of GQDs by the effective orbital resonance effect. The density functional theory calculation demonstrated that the band gap of GQDs can also be tuned and decreased as illustrated in Figure 12e [128]. Furthermore, Jin et al. discovered that two independent molecule-like states existed in GQDs. which were attributed to the surface moieties’ structure, further indicating that surface state can affect PL emission of GQDs. Furthermore, an individual effect is unable to fully explain PL emission of GQDs. The synergistic affect models are proposed for interpretation of PL emission mechanism of CDs. The models include carbon core and surface state, the conjugation effect and surface functional groups, the conjugation effect and defects. The reasonable PL emission mechanisms of CQDs have been discovered and are probably derived from the surface/edge state and conjugated *π*-domains. Nonetheless, Wang et al. suggested that PL behaviors of GQDs were probably associated with oxygen-containing functional moieties on the surface of carbon core by single-particle spectroscopic measurements [129]. The photoexcited electrons relaxed from π* orbit to sp^2^ intrinsic states and the defect states, giving rise to blue and green PL emission, respectively, as schematically shown in Figure 12f. The green PL emission was attributed to relaxation of electrons into the hybrid structure between carbon core and the oxygen-containing functional groups. 

**Figure 13:**
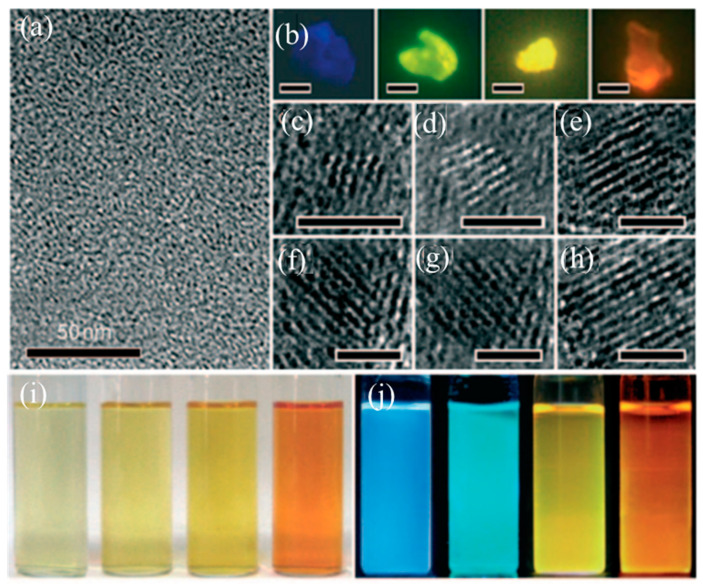
Quantum size effect in carbon quantum dots (CQDs). (**a**) TEM image of CQDs; (**b**) fluorescent microscopy images of CQDs with an excitation wavelength of 360 nm; (**c**–**h**) HRTEM images of typical CQDs with different diameters; typical sized CQDs optical images illuminated (**i**) underwhite (left; daylight lamp) and (**j**) UV light (right; 365 nm) [130]. Copyright 2010, John Wiley and Sons.

Kang et al. proposed a current density-controlled electrochemical route to prepared CQDs with the different diameters. PL emission of CQDs was sensitive to its size as shown in Figure 13 The PL emission wavelengths of the small CQDs were observed in the ultraviolet region, the PL emission wavelengths of the medium sized CQDs were observed in the visible region, and the PL emission wavelengths of large CQDs were observed in the near-infrared emission. Kang et al. further investigate the relationship between PL emission and cluster size of CQDs by theoretical calculations for explaining origin of PL emission of CQDs, indicating that gaps of HOMO–LUMO was dependent on the size of the graphene fragments. Thus, they deduced that the strong emission of CQDs comes from the quantum-sized graphite structure [130].

**Figure 14:**
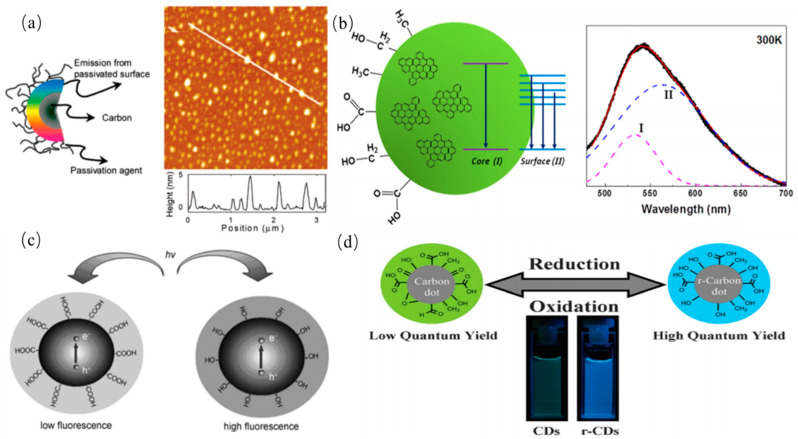
(**a**) Surface state and carbon-core state in CQDs [131]. Copyright 2007, American Chemical Society. (**b**) Dual fluorescence bands observed in CQDs [132]. Copyright 2012, American Chemical Society. (**c**) Graphical comparison about effect of electron-withdrawing and electron-donating surface functional groups on CDs PL [133]. Copyright 2011, Springer Nature. (**d**) Diagram about oxidation/reduction of surface functional groups to alter the luminescence of CDs [134]. Copyright 2011, Royal Society of Chemistry.

The surface functional groups of CQDs possess various energy levels, resulting in a series of emissive traps. The emissive traps of surface states will dominate the PL emission of the CQDs. The more surface functional groups and effective modification can offer more surface defects, which may result in a red-shifted emission. It is worth emphasizing that the surface state consists of the hybridization of the carbon backbone and connected chemical groups. Sun et al. systematically investigated the effect of surface states on PL emission by comparing with CQDs passivated with different solvents, further indicating that surface energy traps controlled the PL emission as shown in Figure 14a [131]. The dual PL bands of CQDs were observed by Tang et al. which was attributed to core and surface state emissions. The dual emission bands exhibited similar temperature dependence with the temperature varying as shown in Figure 14b [132]. The two overlapping spectral PL bands of CQDs were attributed to the intrinsic and extrinsic states. Liu et al. prepared CQDs by using candle soot as a carbon source under alkaline conditions, and investigated the pH response property. The PL emissions of the obtained fluorescent CQDs were quenched under acidic conditions, and were restored under alkaline conditions, which is attributed to destruction of hydroxyl groups on surface states of CQDs under acidic conditions, indicating that hydroxyl-group coatings on CQDs can enhance PL as shown in Figure 14c [133]. Zheng et al. prepared CQDs rich in hydroxyl functional groups by using sodium borohydride, and confirmed that electron-donating surface groups can effectively enhance PL emission of CQDs [134]. Their method significantly increased the number of surface hydroxyl groups without reducing other species, resulting in enhanced CQDs luminescence as shown in 14d [134].

#### 3.1.3. Up-Conversion Photoluminescence Property

**Figure 15:**
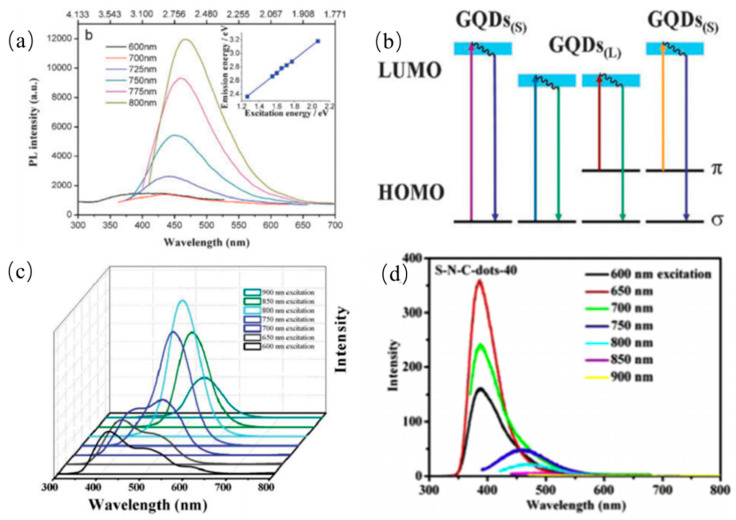
(**a**) The up-converted emission of PEG passivated GQDs, and (**b**) The proposed normal and UCPL emission mechanism of GQDs [135]. Copyright 2011, Royal Society of Chemistry. (**c**) The red-shifts of the up-conversion PL emission of GQDs [136]. Copyright 2012, Royal Society of Chemistry. (**d**) The up-conversion PL properties of the N-S-CQDs [109]. Copyright 2013, Elsevier.

In contrast to traditional PL emission, the certain CQDs can exhibit UCPL emission properties, that is to say, the emission wavelengths of CQDs are shorter than their excitation wavelengths. Two-photon excitation and anti-stokes PL emissions are usually two kinds of the most popular explanations for the up-conversion fluorescence phenomenon. Firstly, Shen et al. fabricated PEG passivated GQDs via acidic oxidation in 2011. The up-converted emission wavelengths were observed in the range of 390 to 468 nm with the excitation wavelength increasing from 600 to 800 nm as shown in Figure 15a, respectively [135]. Remarkably, GQDs showed stable and independent UCPL emission which were attributed to the carbine ground-state multiplicity. The UCPL emissions were elucidated that secondary photoexcited electrons originating from *p* orbital relaxed back to a low energy state. Shen et al. also observed that the CQDs synthesized from walnut shells exhibit up-conversion PL emission which is also called anti-stokes PL emission [135]. Specifically, the energy gap between LOMO and HOMO of CQDs decreases gradually as their sizes increases. These electrons located at HOMO orbital are excited by low energy photons to LOMO orbital. However, the excited electrons at LOMO orbital are instable, and then transit to the σ ground-state in the form of irradiation as shown in Figure 15b. In the other report, the red-shifts of the up-conversion PL emissions of GQDs were observed with excited wavelengths increasing from 600 nm to 900 nm, which was elucidated to be the multi-photon active process as presented in Figure 15c [136]. Sun’s group also proposed a multi-photon active process mechanism to elucidate UCPL emission of the S-N-CDs produced from hair fiber via ultrasonic-assisted treatment as shown in Figure 15d [109]. The as-prepared S-N-CDs are known as an efficient two-photon fluorescent probe for in vivo imaging, especially in biological tissue imaging. Moreover, Zhuo et al. reported excitation-independent up-conversion PL emission [55].

### 3.2. Photoinduced Electron Transfer (PET) Property

**Figure 16:**
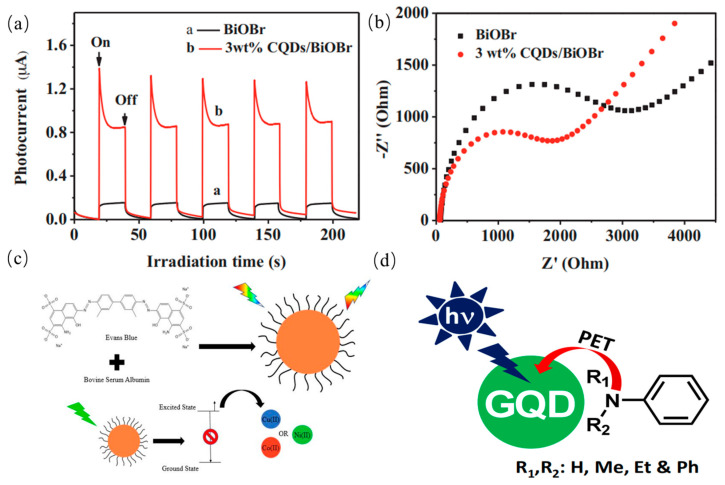
Transient photocurrent response (**a**) and electrochemical impedance spectra (**b**) of pure BiOBr and the CQDs/BiOBr composite [138]. Copyright 2016, Elsevier. (**c**) The PET process between amine functional groups and metal ions interacted [139]. Copyright 2018, American Chemical Society. (**d**) The PET processes between GQDs and various aniline derivatives [140]. Copyright 2015, American Chemical Society.

The photoinduced electron transfer of the CQDs has received extensive investigations in light-energy conversion field. Sun and coworkers observed that PL emission of CQDs in solution were efficiently quenched while either electron acceptors or electron donors were present. Until now, there has not been direct evidence with photoinduced electron separation of CQDs. Nonetheless, some studies have been interpreted as some indirect experimental proof of photoinduced electron separation of CQDs by redox processes. Kang’ group reported CQDs have photoinduced electron transfer properties including either electron acceptors or electron donors by PL decay method [137]. It was demonstrated that photogenerated electrons of semiconductors can be efficiently trapped by CQDs-based composite photocatalysts by Xia et al. [138], further improving separation efficiency of photoinduced carriers as show in Figure 16a,b. Moreover, Xu et al. found that noble metal can deposit on the CQDs surface upon photoexcitation. The disruption of photo-induced carriers can result in near-neighbor static quenching, leading to efficient static quenching of fluorescence emissions. Keenan et al. prepared CQDs with long-wavelength PL emission which was associated with the amine sites [139]. PL emission of carbon dots was quenched accordingly when metal ions interacted with amine functional groups. The phenomenon was considered that the electron from an amine group was transferred to a metal cation in proportion to the reduction potential of the metal by non-radiative route as presented in Figure 16c. Ghosh et al. systematically reported the PET processes between GQDs and various aniline derivatives. These derivatives as electron donors were utilized to detect radical cations [140]. PL emission of GQDs was significantly quenched when the GQDs interacted with the aniline derivatives in the excited state as shown in Figure 16d, indicating that GQDs can be candidates for understanding the mechanism of PET for various donor–acceptor systems. 

### 3.3. Catalytic Properties

**Figure 17:**
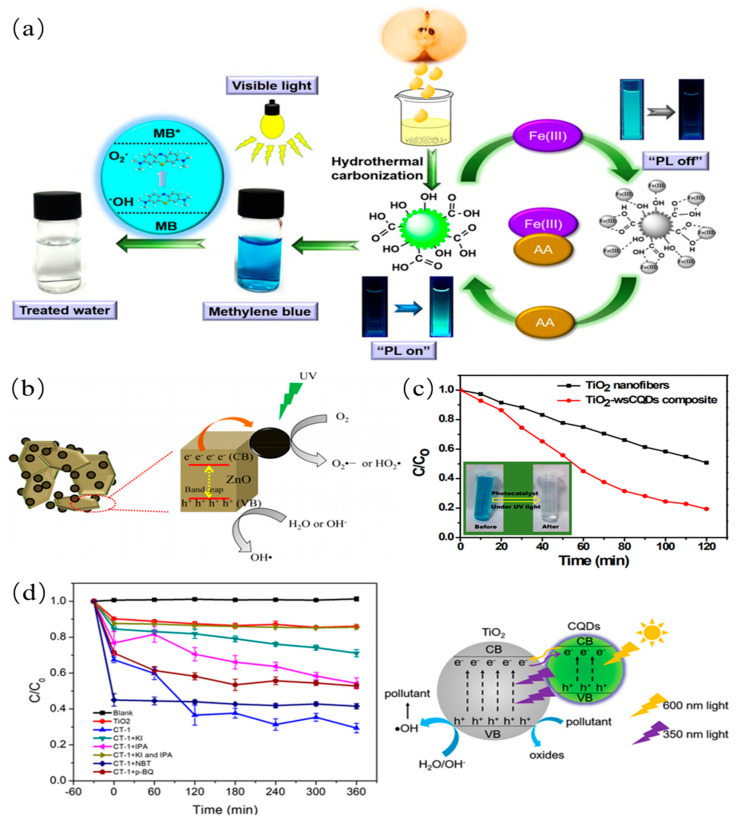
(**a**) Schematic of the as-synthesized CQDs from pear juice and their visible-light-induced dye degradation [141]. Copyright 2019, Springer Nature. (**b**) Illustration of photocatalytic process of CQDs/ZnO composite catalysts [34]. Copyright 2013, American Chemical Society. (**c**) Photocatalytic degradation of methylene blue by CQDs/TiO_2_ composite [38]. Copyright 2016, Royal Society of Chemistry. (**d**) Catalytic performance and mechanism of a free radical scavenger [142]. Copyright 2021, American Chemical Society.

Due to an excellent photocatalytic activity, CQDs were widely applied in photocatalytic field for improving activity of catalysts. For instance, Kim et al. synthesized the visible-light-responsive CQDs from pear juice for the efficient degradation of methylene blue as shown in Figure 17a [141]. The superior photocatalytic activity was attributed to efficient light absorption and electron transfer of CQDs. The degradation ratio of MB reached 95% within 130 min. Prasannan et al. proposed a route to synthesize C-dots/ZnO composite catalyst by loading CQDs produced from orange peels waste on ZnO [34]. The photocatalytic activity of C-dots/ZnO composite catalyst is higher than that of pure ZnO catalyst as shown in Figure 17b. In this process, the excited electrons from ZnO are transferred to CQDs, preventing the recombination of electron–hole pairs. And then, hydroxyl radicals were produced from interaction between ZnO and water. Meanwhile, superoxide ions were generated from interaction between electrons on CQDs and oxygen. The obtained superoxide ions can be utilized to degrade azo dye. The synthesized CQDs from lemon peel waste are immobilized on TiO_2_ to produce CQDs/TiO_2_ composite catalyst by Tyagi et al. [38]. The obtained CQDs/TiO_2_ catalyst was used for the degradation of methylene blue dye as shown in Figure 17c. These results suggest that CQDs play a key role in the photocatalytic field. Furthermore, Deng et al. also synthesized CQDs/TiO_2_ composites for the efficient degradation of methyl orange as shown in Figure 17d [142]. The degradation ability was attributed to the existence of $h^{+}$ and •OH under visible light. These results mean that the ever-increasing water deterioration issues can be hopefully solved in the further.

## 4. Applications of CDs

As high-efficiency fluorescent materials, CDs have many remarkable advantages including tunable PL emission, good biocompatibility, low toxicities, and outstanding photoinduced electron transfer therefore show great potential for various applications in sensors, bioimaging, solar cells and photocatalysis, etc., Therefore, we will discuss the use of CDs in various field in the following sections.

### 4.1. CDs in Sensing

As one of the important applications of CDs, fluorescent sensors have been utilized in detection of metal ions, anions, and molecules. PL emission of CDs was quenched by the ions or molecules owing to photo-induced charge transfer and resonance energy transfer. Compared with other ions, Fe^3+^ is the most commonly detected ion owing to its effective combination with CDs. In addition, some other anions such as ClO^−^, S^2−^ and PO_4_^3−^ can be detected by PL emission signal quench of CQDs. Furthermore, some molecules can be sensitively detected by electrostatic interaction with CDs. 

#### 4.1.1. CDs in Sensing of Metal Ions

**Figure 18:**
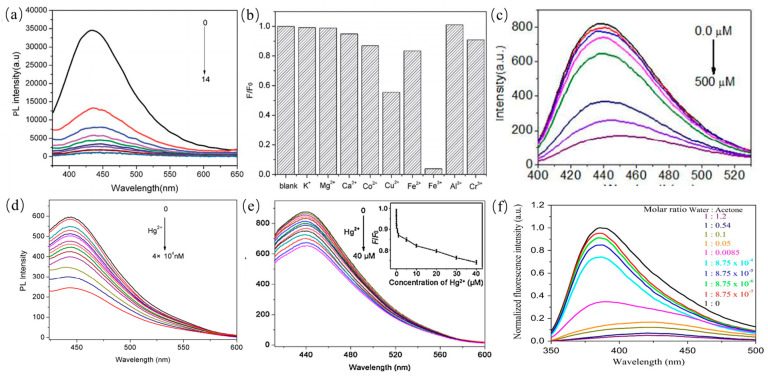
(**a**) The PL emission of S-CDs for detection of Fe^3+^ ions at different concentrations, and (**b**) The bar chart of the responses of S-CQDs [143]. Copyright 2015, Royal Society of Chemistry. (**c**) PL emission of CDs for detection of Fe^3+^ ions [144]. Copyright 2015, Royal Society of Chemistry. (**d**) PL spectra of CDs in the presence of various concentrations of Hg^2+^ [78]. Copyright 2012, American Chemical Society. (**e**) PL emission spectra of CDs upon addition of various concentrations of Hg^2+^ [149]. Copyright 2013, Elsevier. (**f**) Fluorescence spectral profile of CD in different molar ratio of water and acetone [150]. Copyright 2016, Elsevier.

As mentioned above, Fe^3+^ ions, are considered as the most commonly detected ions in the field of sensors. PL emission signal of CDs quenches when iron ions combine with the surface functional groups such as amino moieties. For example, Liu et al. synthesized CQDs from goose feather for sensitive and selective detection of Fe^3+^ ions with a low detection limit as shown in Figure 18a,b [143]. Xu et al. also reported detection of Fe^3+^ ions by using CDs. It was clearly observed that PL emission intensity of CDs decreased monotonically with the addition of Fe^3+^ ions as shown in Figure 18c, suggesting that Fe^3+^ ions can quench PL signals of CDs owing to strong coordination between Fe^3+^ ions and amine-rich groups on the surface of CDs [144]. In addition to iron ions, some other ions such as Hg^2+^ and Pb^2+^ can also be detected in water for the environmental pollution [145,146,147,148]. Liu and coworkers reported the preparation of CDs from pomelo peel waste as carbon source for Hg^2+^ detection in tap, lake and river water with a low detection limit, which was based on PL quenching of CDs as shown in Figure 18d [78]. Some researchers also utilized flour, sweet potato or cucumber juice as low-cost carbon sources to prepare CDs for the detection of Hg^2+^, which showed excellent detection limit of priority heavy metal ion or acetone contaminants of environmental interest as shown in Figure 18e,f [149,150]. 

#### 4.1.2. CDs in Sensing of Anions

**Figure 19:**
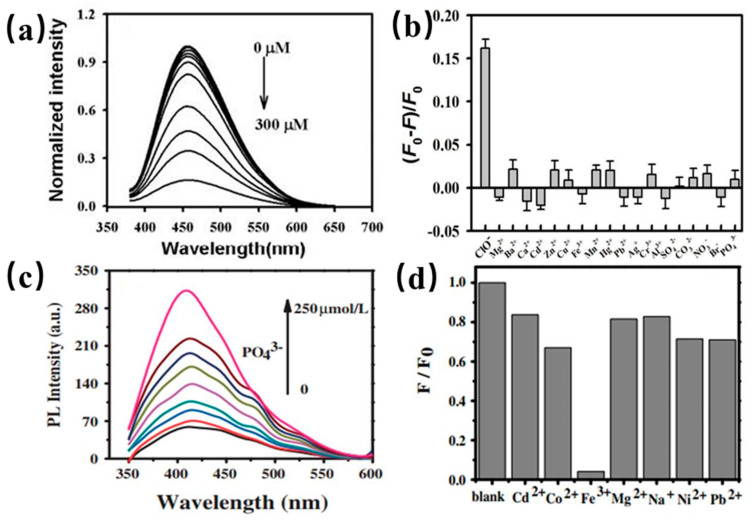
(**a**) FL response of BCDs dispersion in the presence of various concentrations of NaClO, and (**b**) Selectivity of the BCDs-based sensor for hypochlorite solution [110]. Copyright 2013, Royal Society of Chemistry. (**c**) PL emission of BCDs in the presence of various concentrations of PO_4_^3−^, and (**d**) selectivity of the BCDs with different ions [151]. Copyright 2014, John Wiley and Sons.

In addition to metal ions, PL emissions of CDs were also utilized to detect anions such as ClO^−^, PO_4_^3−^ and S^2−^ [110,151,152]. As shown in Figure 19a,b, PL emission quenching of CDs dispersed in ClO^-^ was reported by Yin et al. indicating that ClO^−^ combines with the functional groups on the surface of CDs [110]. Therefore, the selective detection of the ClO^−^ anion can be achieved by surface modification of CDs. Xu and coworkers showed that boron doped CDs from potatoes were utilized to sensitively detect PO_4_^3−^ with a detection concentration limit of 0.8 µM as shown in Figure 19c,d [151]. The PL emission of B-CDs is quenched significantly when PO_4_^3−^ anions were in the solution. Jin et al. reported that BCDs derived from natural carrots were used to detect S^2−^ with a low detection limit of 0.06 µM. The boron doped CDs showed the radiometric two-photon PL emission for turn-on response to S^2−^ [152]. Thus, CDs were considered as a probe for detection of anions with a low concentration limit. 

#### 4.1.3. CDs in Sensing of Molecules

**Figure 20:**
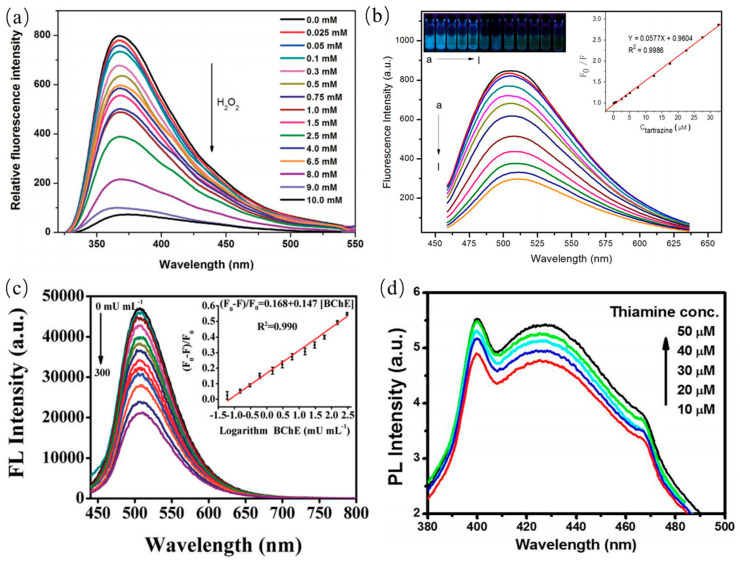
FL response of CDs dispersion in the presence of various molecules: (**a**) H_2_O_2_ [153]. Copyright 2014, Royal Society of Chemistry. (**b**) Tartrazine [156]. Copyright 2015, American Chemical Society. (**c**) Butyryl cholinesterase [155]. Copyright 2018, Royal Society of Chemistry. (**d**) Thiamine [157]. Copyright 2016, Elsevier.

PL properties of CDs can also be widely used to detect various molecules such glucose and H_2_O_2_ [153], pyridine, dopamine [154], catechol, cholinesterase [155] etc. For instance, Shan et al. reported the PL emission quenching of CDs in the presence of H_2_O_2_ within concentration range of 0.1 to 1.0 mM as shown in Figure 20a, which was elucidated that the charges transfer between boron and H_2_O_2_ [153]. Xu and coworkers synthesized BCDs for the detection of the Tartrazine molecule as presented in Figure 20b [156]. PL emission of BCDs was quenched as the Tartrazine increasing from 0.25 µM to 32.5 µM. In other work, nitrogen-doped CDs were employed to sensitively detect the glucose molecule with a detection range of 1–12 mM. Song and coworkers prepared CDs from natural precursor for the sensitive detection of butyryl cholinesterase (BChE) as shown in Figure 20c [155]. The corresponding detection limit of BChE is 0.035 mU/mL. Thus, CQDs have great potential to be used as an indicator for detecting nerve gases and can be applied in the field of biosensors. Purbia et al. prepared the boron doped CDs for sensitively detecting thiamine with an error of less than 3% as shown in Figure 20d [157]. The PL emission of BCDs was quenched in the presence of thiamine, which was elucidated that BCDs electrostatically interact with the surface charge of the thiamine. 

### 4.2. CDs in Bioimaging

**Figure 21:**
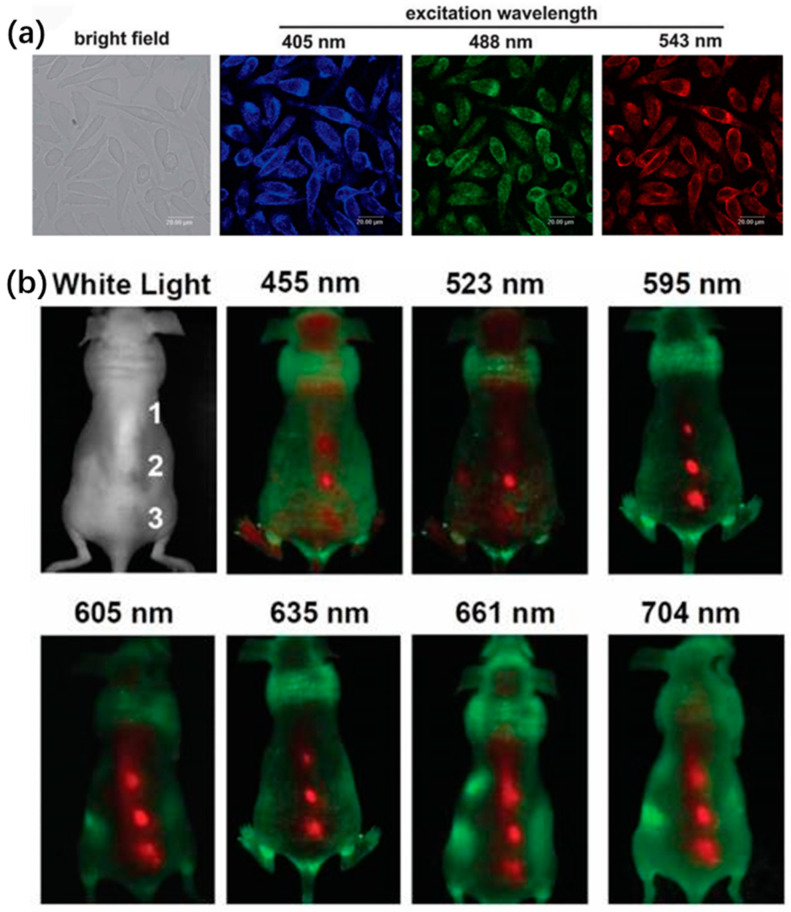
(**a**) The in vivo images of L929 cells which are injected with CDs under different excitation wavelengths [163]. Copyright 2012, Royal Society of Chemistry. (**b**) The in vivo fluorescence images of CDs that are injected into a nude mouse [164]. Copyright 2011, John Wiley and Sons.

Due to low/non-toxicity of CDs, they have also been considered as biocompatible fluorescent dyes for in vivo imaging instead of carrier of drug molecules. The CDs derived from different carbon sources are not abided photobleaching and photodegradation. As a superior candidate, CDs can be modified with various functional groups for appropriate PL emission. In the past years, a number of studies have reported the bioimaging potentials of CDs [158,159,160,161]. The PL emission of CDs was commonly tuned to a longer wavelength range for improving signal to noise ratio (SNR). That is to say, PL emission of CDs in the NIR region was specifically critical and significant for in vivo optical imaging due to the tissues background in the NIR “water window”. Compared to other heavy metal QDs, the CDs with strong absorptivity can compensate lower fluorescence yield for bioimaging. Sun et al. first proposed a route to stain Caco-2 cells by using the PEG1500N passivated CDs for cellular imaging, suggesting CDs can be utilized in fluorescent labels of cell [162]. Subsequently, some other cells such as HeLa cells, HepG2 cells, MCF-7 cells, pancreas progenitor cells and human lung cancer cells were also used in intracellular imaging. Zhai et al. reported that L929 cells incubated with CDs show intense and stable blue, green and red PL emission with the excitation wavelengths of 405 nm, 488 nm and 543 nm as presented in Figure 21a [163]. The CQDs were injected into a nude mouse at three different locations and fluorescent images were collected with different excitation lights, whereas only red spots were observed in the in vivo fluorescence images of CDs as shown in Figure 21b [164]. Wei et al. prepared N-S-BCDs from allium fistulosum for cellular multicolor imaging owing to their low cytotoxicity. The surface of CDs was commonly modified with appropriate functional groups for improving absorptivity with small molecules or proteins [165]. For example, CDs passivated with a hyperbranched polymer showed stronger PL emission than that coated with a linear polymer. 

### 4.3. CDs in Drug Delivery

**Figure 22:**
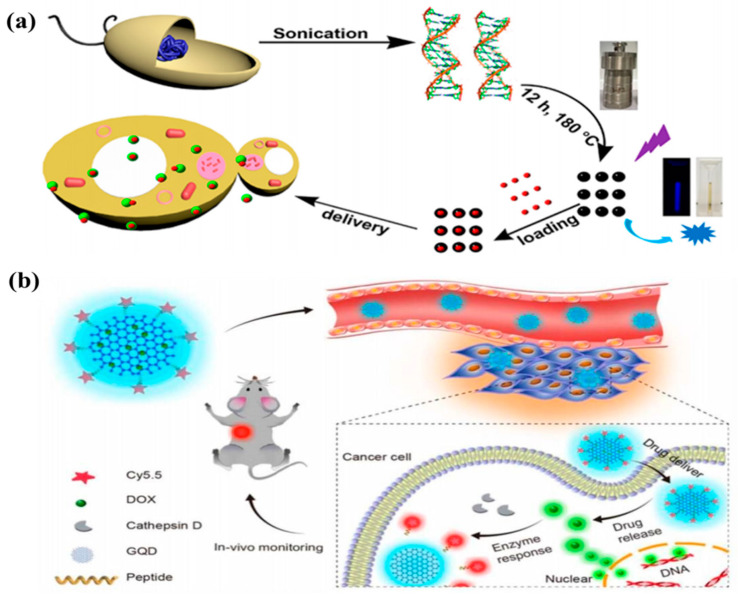
(**a**) Schematic image of DNA-BCD synthesis and application in drug delivery [166]. Copyright 2015, American Chemical Society. (**b**) Strategy of CD-based theranostic agent in vivo monitoring [167]. Copyright 2017, American Chemical Society.

CDs were considered as multifunctional vehicle for drug delivery systems owing to intense fluorescent, low toxicity, chemical inertness and excellent biocompatible. The specific drug was transported and eliminated at a precise target for therapy. Recently, CDs have been a focus on drug delivery due to their superior properties. For example, Karthik et al. loaded drug to nitrogen-doped CDs by a covalent connection. The aggregation of the drug-loaded CDs was transported to the cytoplasm and nucleus of cancer cells. The drug was released at the specific sites by means of irradiation. Ding and coworker synthesized BCDs by using genomic DNA as a carbon source for studying drug delivery as shown in Figure 22a [166]. In addition, CDs with micro/nanopore structures have received increasing attention for drug delivery owning to their efficacy. The common drug doxorubicin was loaded on micro/nanopore CDs. The CDs-DOX was released in A549 cells by controlling pH. Ding et al. designed a DOX loaded CD-based theranostic agent for drug delivery [167]. The internalization of the nanoagent was tracked by PL emission signal from CDs as shown in Figure 22b. The blue PL emission was turned to the green PL emission of DOX after release of DOX in tumor tissue for substantial killing of cancer cells. In the other experiment, CDs coupled with Au nanoparticles were conjugated with PEI–pDNA for drug delivery. The quenching fluorescence emissions of Au-CD can show the release of pDNA in the cell cytoplasm, further suggesting that Au-CDs have significant transfection efficiency. 

### 4.4. CDs in Photocatalysis

Now, many researchers have focused on these two major issues including environmental pollution and energy resource crisis. In order to solve these problems, researchers proposed a “green” and potential route for generating energy and reduction of environmental pollutants by means of photocatalysis [168,169,170,171,172,173,174,175]. We will introduce the recent applications of CQDs-derived photocatalysts in dye degradation, solar water splitting as well as CO_2_ conversion in this section. The CQDs-derived photocatalysts were modified by immobilized and loaded method for the development of the photocatalytic performance. A brief summary of recent progress on CDs-derived photocatalysts are given in Table 3.

#### 4.4.1. Photocatalytic Degradation

**Figure 23:**
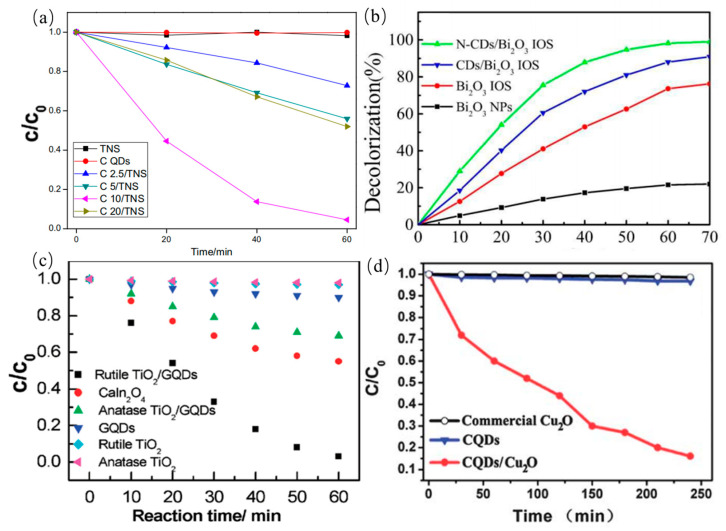
Degradation of RhB by (**a**) CQDs/TNS composites photocatalyst [168]. Copyright 2014, Elsevier. (**b**) N-CDs/Bi_2_O_3_ composite photocatalyst; degradation of MB [169]. Copyright 2015, Royal Society of Chemistry. (**c**) CDs/rutile TiO_2_ composite photocatalyst [55]. Copyright 2012, American Chemical Society. (**d**) GQDs/Cu_2_O composite photocatalyst [170]. Copyright 2012, Royal Society of Chemistry.

As a promising candidate, the doped CDs possess unique fluorescence behavior and photoelectron transfer properties for high-performance photocatalyst. The surface functional groups of CDs are adjusted and their band gaps are decreased, further facilitating the electron transfer among reactions. Advanced CQDs-derived photocatalyst materials for the degradation of dyes are central to the area of the environmental pollution. The CQDs-modified TNS composites (CQDs/TNS) were prepared by mutual electrostatic interactions for the degradation of the RhB by light irradiation [168]. Compared with pure TNS or CQDs, CQDs/TNS exhibited higher photocatalytic activities. In addition, photocatalytic activity of CQDs/TNS photocatalyst are even higher than CQDs/P25 composites as shown in Figure 23a. Meanwhile, Sun et al. synthesized N-CDs/Bi_2_O_3_ composite photocatalyst for the degradation of RhB [169]. They found that the obtained N-CDs/Bi_2_O_3_ have higher photocatalytic efficiency than CDs/Bi_2_O_3_, Bi_2_O_3_ and Bi_2_O_3_ NPs as shown in Figure 23b, indicating that N-doped CDs are beneficial to improve the absorption intensity of light and electronic transmission properties. Similarly, Zhuo et al. reported the CDs/rutile TiO_2_ composite photocatalyst for degradation of MB. The composite photocatalytic efficiency of the photocatalyst is nine times higher than that of the CDs/anatase TiO_2_ by surface photovoltage measurement. Little reduction of pure rutile TiO_2_, pure anatase TiO_2_, or pure CDs as the catalysts was observed in the control experiments, indicating that CDs/rutile TiO_2_ composite has the excellent photocatalytic activity as shown in Figure 23c [55]. Another photocatalyst, GQDs/Cu_2_O composite, was fabricated for photocatalytic degradation of MB by Li group [170]. GQDs/Cu_2_O composite photocatalyst can develop the photocatalytic degradation efficiency of up to 90%, indicating that GQDs/Cu_2_O composite was beneficial to the photocatalytic degradation of MB as shown in Figure 23d. For comparison, control experiments were further carried out using only pure GQDs or Cu_2_O as photocatalysts, and little reduction of MB was observed in the control experiments. The GQDs/Cu_2_O composite truly realized the efficient usage of the full spectrum of sunlight. In addition, MO, the gas-phase benzene and methanol were also degraded by different CDs derived composite photocatalysts. The obtained photocatalysts showed high photocatalytic activity. 

#### 4.4.2. Solar Water Splitting

The combustion of fossil fuel not only causes environmental pollution but also leads to the greenhouse effect. Hence, worldwide researchers are urgently searching for a kind of clean and renewable energy to replace fossil fuel [171]. Recently, researchers found H_2_ as an ideal energy substitute for fossil fuel due to renewability and high energy density. In general, H_2_ fuels are derived from water splitting. As the promising and efficient approaches, PEC water splitting and photocatalytic water splitting attract widespread attention in the application of stockpile and converses solar energy. The overall water splitting processes involves two half- processes. One is the H_2_-evolution reaction (HER) and the other is the O_2_-evolution reaction (OER). The typical overall reaction equations are given as follows.

(1) $$\mathrm{The}\;\mathrm{overall}\;\mathrm{water}\;\mathrm{splitting}\;\mathrm{processes}:\;H_{2}O\left(l\right)\to O_{2}\left(g\right)+2H_{2}\left(g\right)$$

(2) $$H_{2}\mathrm{-evolution}\;\mathrm{reaction}:\;4H^{+}+4e^{-}\to 2H_{2}\left(g\right)$$

(3) $$O_{2}\mathrm{-evolution}\;\mathrm{reaction}:\;2H_{2}O\left(I\right)\to O_{2}\left(g\right)+4H^{+}+4e^{-}$$

To enhance water splitting efficiency, it is very necessary to seek for a kind of catalyst with high efficiency. CDs have been considered as new-generation advanced photocatalysts materials for H_2_ evolution. In addition, CDs are considered as a kind of light absorber to broaden light absorption range, indicating that they promisingly play a key role in water splitting. 

**Figure 24:**
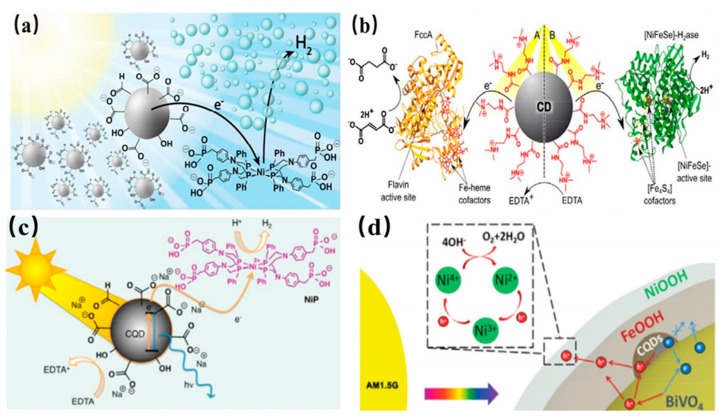
(**a**) Representation of the hybrid CQD-NiP assembly as photosensitizers for H_2_ evolution [172]. Copyright 2015, American Chemical Society. (**b**) Studied of the two independent photocatalytic systems [173]. Copyright 2016, American Chemical Society. (**c**) Representation of the hybrid CQD-NiP system for solar H_2_ production [172]. Copyright 2015, American Chemical Society. (**d**) Representation of the PEC water oxidation using CQDs at the photoanode [175]. Copyright 2017, Royal Society of Chemistry.

Recently, water splitting has been achieved by CQDs/BiVO_4_ composites photocatalysts [172]. Compared with pure BiVO_4_ QDs, the composite CQDs/BiVO_4_ photocatalysts exhibited higher H_2_ evolution rate. The homogeneous CQDs/NiP photocatalytic hybrid system was utilized by sacrificial electron donors for H_2_ evolution. In photocatalytic hybrid system, CQDs and NiP molecules have different functions, that is to say, that CQDs and NiP molecules were regarded as photosensitizers and catalyst, respectively, as shown in Figure 24a. The photoexcited electrons produced from CQDs by UV-vis light irradiation were transferred to solution. The CQDs and NiP can be assembled into photocatalytic hybrid system with sacrificial electron donors for further H_2_ generation. As one of the new photosensitizers, CDs show many remarkable advantages, such as low-cost, nontoxicity, chemical versatility, and outstanding photoinduced electron transfer, making them promising candidates for biological systems in H_2_ evolution. Especially amine terminated CDs show higher catalytic activity than pure CDs, indicating that the surface-bound amine groups of CDs are beneficial to interfacial interactions with other molecules like FccA or H_2_ ase as shown in 24b [173]. This is to say that the surface-bound amine possesses a highly catalytic activity in the H_2_ evolution reaction. Compared with negatively charged CDs, positively charged ammonium-terminated CDs-NHMe^2+^ displayed excellent interfacial interactions and efficient direct electron transfer property, demonstrating that photocatalytic H_2_ generation with H_2_ ase and light driven C=C bond hydrogenation with FccA [174]. The homogeneous CQDs/NiP photocatalytic system, including the photosensitizer CQDs and the catalyst NiP molecule was used for H_2_ production as shown in shown in Figure 24c [172]. In order to sacrificial electron donor, CQDs can transferred photoexcited electrons to solution under UV and visible light by dissolving CQDs and NiP in buffer solution, result in the catalytic system with an activity. Moreover, PEC water splitting showed high activity at the NFCB photoanode for H_2_ production as shown in Figure 24d [175]. Compared with the pristine BiVO_4_ photoanode, the NFCB photoanode presented a remarkable photocurrent density.

#### 4.4.3. CO_2_ Conversion

**Figure 25:**
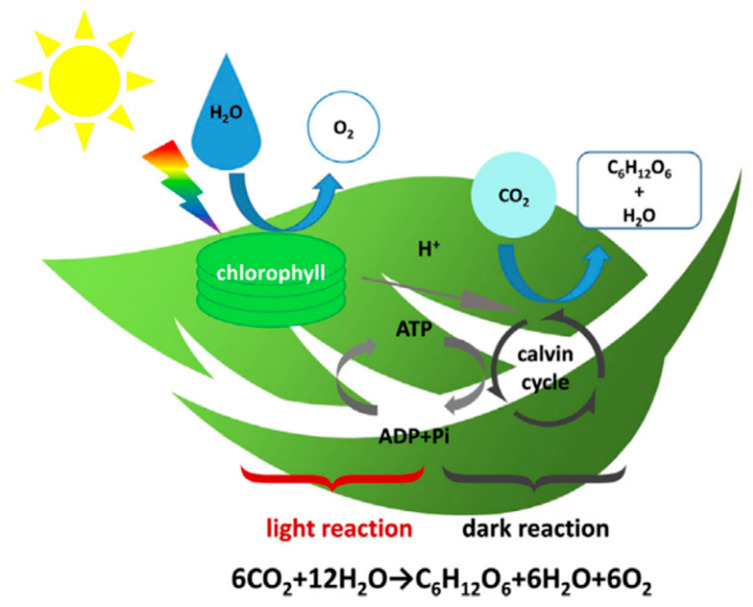
A schematic illustration of photosynthesis [176]. Copyright 2018, Elsevier.

The excessive emission of CO_2_ results in greenhouse effect, so there is a strong demand for converting CO_2_. Photosynthesis, as an effective conversion method, is divided two processes including light reaction and dark reaction as shown in Figure 25 [176]. It is well known that green plants and algae, as efficient processing plants, can absorb CO_2_ and release O_2_ by the photosynthesis. Similarly, many researchers proposed to produce an “artificial leaf” by imitating photosynthesis for CO_2_ conversion. However, noble metal catalysts have shortcomings of high-cost, low selectivity and high energy input, which further impede their application in the field of CO_2_ conversion. As a befitting substitution, CDs can efficiently convert CO_2_ into non-polluting products in photocatalytic process. Wang et al. found that CO_2_ gas in the purged solution reduce by visible light irradiation for 5 h after Pt or Au covering on the PEGylated CDs were added to optical cells for CO_2_ conversion, indicating that CO_2_ have been converted in this process [109]. Pathak et al. reported that PEG1500N-functionalized CQDs coated by Au can convert CO_2_ into acetic acid and formic acid by the visible-light illumination. In general, R value is used to evaluate the performance of CQDs-base composite catalysts for CO_2_ photoreduction. In fact, CDs-based catalysts have not been extensively investigated for CO_2_ conversion. Hence, researchers shall pay more attention to this field of CO_2_ conversion for solving greenhouse effect and producing new energy as soon as possible.

## 5. Conclusions and Future Outlook

In this review, we mainly focus on recent advance in synthesis of CDs, optical properties and their applications, especially in the fields of biosensors, bioimaging, drug delivery, and photocatalysis. Various synthesis routes have been critically reviewed, and they have advantages including being low-cost and having easy availability and large-scale production. The synthesized CDs show many remarkable advantages including low cytotoxicity, efficient light harvesting, and outstanding photoinduced electron transfer, making them efficient and versatile materials for biosensors, in vitro bioimaging, drug delivery, and photocatalysis. 

Even if researchers have made great efforts, there remain many challenges in the synthesis of multifunctional CDs with a specified structure. The controllable preparation of CDs is a crucial issue to be solved due to the many variables involved, including the nature of reaction precursors, reaction conditions, and quantitative reaction indicators. In addition, the PL mechanism of CDs are ambiguous and further hinders their applications in many other areas such as bioimaging and drug delivery. Fluorescence of CDs is particularly important for expanding the applications of CDs in near infrared imaging. The blood circulation and toxicity of CDs are still further evaluated for simultaneous bioimaging and drug delivery. Besides, controllable preparation of CDs with desired high QY is still a big challenge. Precisely controlled surface functionalization of CDs is beneficial to study of PL mechanism and their broad application. Works related to puzzle and self-assembly of CDs to 2D/3D morphology are rare, and there is almost no work regarding fluorescent mechanism research of assembled materials. The macroscopic particle behaviors are fantastic and deserved to be studied further. Moreover, ultraviolet absorption of most CDs limits their application, especially in bioimaging. Hence, the preparation of CDs with longer wavelengths will become a research hotspot. Despite these tough works above are still remaining chronically, great progress has already been achieved in CDs field nowadays. The future of CDs presents a good vision in fundamental research and practical applications.

## Figures and Tables

**Table 1:** Preparation of CDs from various biomass carbon sources and their applications.

Source	Synthesis Condition	Application	Ref.
Wheat straw	250 °C, 10 h	imaging and sensing	[67]
Coffee grounds	200 °C, 6–10 h	detection of Fe^3+^ and Cu^2+^	[84]
Onion waste	120 °C, 2 h	detection of Fe^3+^ and imaging	[85]
Wheat bran	180 °C, 3 h	drug delivery	[86]
Tobacco leaves	200 °C, 3 h	detection of tetracyclines	[87]
Orange peels	120 °C, 12 h	photocatalysis	[34]
Coconut husks	200 °C, 3 h	detection of Hg^2+^	[88]
Tulsi leaves	180 °C, 4 h	detection of Pb^2+^	[89]
Prawn shell	180 °C, 12 h	detection of nitrite	[90]
Rice residue	200 °C, 12 h	detection of Fe^3+^ and tetracyclines	[91]
Magnolia flower	200 °C, 8 h	detection of Fe^3+^	[81]

**Table 2:** Comparison of the merits and demerits of representative CDs.

Synthetic Methods		Merits	Demerits	Ref.
Top-Down	Chemical exfoliation	Most accessible, various sources	Harsh conditions, drastic processes, multiple-steps, poor control over sizes	[39,40,41,42,43,44,45,46,47,48,49]
	Laser ablation	Fast, effective, highly tunable	Low quantum yield, poor control over sizes, modification is necessary.	[50,51,52,53,54]
	Ultrasonic-Assisted treatment	Easy operation	Instrumental wastage, high energy cost	[54,55]
Bottom-up	Microwave synthesis	Fast, scalable, inexpensive, eco-friendly	Poor control over sizes	[60,61,62,63,64]
	Hydrothermal	Inexpensive, eco-friendly, non-toxic	Poor control over sizes	[66,67,68,69,70,71,72,73,74,75,76,77,78,79,80,81]
	Solvothermal	Inexpensive, eco-friendly, non-toxic	Poor control over sizes	[93,94,95,96,97,98,99,100,101,102,103]
	Pyrolysis/Carbonization	Easy operation, solvent-free, low-cost, large-scale production	Non-uniform size distribution	[104,105,106,107,108,109,110,111]
	Chemical vapor deposition	Controllable morphology and size, high yield	Complicated operation, high cost	[112,113,114,115]

**Table 3:** CDs-derived photocatalysts in the different application fields.

Photocatalysts	Applications	Ref.
CQDs/TiO_2_	degradation of MB	[170]
CQDs/SiO_2_	degradation of MB	[171]
CND/Fe-NF	degradation of MO	[55]
CQDs/HQC/TiO_2_	degradation of phenol	[172]
CQDs/P25	water splitting	[171]
CDs/g-C3N4	water splitting	[173]
CQD-NiP	water splitting	[174]
CQDs/Cu_2_O	CO_2_ conversion	[175]

## Data Availability

Data presented in this manuscript is available from corresponding author upon reasonable requests.
